# Predicted short and long-term impact of deworming and water, hygiene, and sanitation on transmission of soil-transmitted helminths

**DOI:** 10.1371/journal.pntd.0006758

**Published:** 2018-12-06

**Authors:** Luc E. Coffeng, Susana Vaz Nery, Darren J. Gray, Roel Bakker, Sake J. de Vlas, Archie C. A. Clements

**Affiliations:** 1 Department of Public Health, Erasmus MC, University Medical Center Rotterdam, Rotterdam, The Netherlands; 2 Research School of Population Health, College of Medicine, Biology and Environment, The Australian National University, Canberra, Australian Capital Territory, Australia; Royal Veterinary College, UNITED KINGDOM

## Abstract

**Background:**

Regular preventive chemotherapy (PCT) targeting high-risk populations is an effective way to control STH in the short term, but sustainable long-term STH control is expected to require improved access to water, sanitation, and hygiene (WASH). However, experimental studies have not been able to conclusively demonstrate the benefit of WASH in preventing STH (re-)infections. We investigated the impact of WASH on STH infections during and after PCT using mathematical modelling.

**Methods and findings:**

We use the individual-based transmission model WORMSIM to predict the short and long-term impact of WASH on STH transmission in contexts with and without PCT. We distinguish two WASH modalities: sanitation, which reduces individuals’ contributions to environmental contamination; and hygiene, which reduces individuals’ exposure to infection. We simulate the impact of varying levels of uptake and effectiveness of each WASH modality, as well as their combined impact. Clearly, sanitation and hygiene interventions have little observable short-term impact on STH infections levels in the context of PCT. However, in the long term, both are pivotal to sustain control or eliminate infection levels after scaling down or stopping PCT. The impact of hygiene is determined more by the effectiveness of the intervention than its overall uptake, whereas the impact of sanitation depends more directly on the product of uptake and the effectiveness.

**Interpretation:**

The impact of WASH interventions on STH transmission highly depends on the worm species, WASH modality, and uptake and effectiveness of the intervention. Also, the impact of WASH is difficult to measure in the context of ongoing PCT programmes. Still, we show a clear added benefit of WASH to sustain the gains made by PCT in the long term, such that PCT may be scaled down or even stopped altogether. To safely stop or scale down PCT, policy for WASH and PCT should be integrated.

## Author summary

Soil-transmitted helminths (STH), which are transmitted via faecal contamination of the environment, still infect over 1 billion people in developing countries because of absence or poor access to improved sources of water, sanitation, and hygiene (WASH). In this study, we use a mathematical model for transmission and control of STH to investigate to what extent and on what time scale we can expect improved access to WASH to impact STH infection levels. Our predictions confirm findings from experimental studies that in the context of deworming campaigns, the impact of WASH is difficult to measure, which is due the strong and quick impact of deworming masking the slower, more long-term impact of WASH. We further show that the impact of WASH interventions on STH transmission highly depends on the worm species, WASH modality, uptake, effectiveness, and pre-control endemicity. Still, we show a clear added benefit of WASH to sustain the gains made by PCT in the long term, such that PCT may be scaled down or even stopped altogether. To safely stop or scale down PCT, policy for WASH and PCT should be integrated.

## Introduction

Globally, over 1 billion people are infected with soil-transmitted helminths (STH) [1], the majority of whom are infected with *Ascaris lumbricoides* (roundworm), *Trichuris trichiura* (whipworm), and/or hookworm (*Necator americanus* and *Ancyclostoma spp*.). STH are transmitted through the ingestion of soil contaminated with egg-containing faeces (roundworm and whipworm) or exposure of skin to free-living larvae (hookworm). As such, prevalence and intensity of STH infections are strongly inversely correlated with access to and use of improved sources of water, sanitation, and hygiene (WASH) [2,3]. World Health Organization (WHO) guidelines recommend that STH are controlled by preventive chemotherapy (PCT) with albendazole (ALB) or mebendazole (MEB) targeted at school age children (SAC), pre-school age children, and groups at high risk of morbidity such as women of childbearing age. The guidelines further recommend that complimentary WASH interventions are implemented to sustain control [4–6]. Although WASH interventions are expected to help interrupt STH transmission such that PCT can be stopped in the long run, it is unclear at what minimum uptake and effectiveness of WASH and within what time frame we can expect this to happen.

There have been several studies attempting to demonstrate and quantify the impact of different WASH interventions on STH infections. So far, several randomised controlled trials (RCT) implementing WASH programmes in schools have reported a decrease in STH infections as a result of different individual or combined WASH components, with a strong emphasis on individual hygiene behaviours [7–10]. In contrast, community-based RCTs implemented in India alongside the Indian Total Sanitation Campaign did not detect any benefit resulting from the sanitation intervention, although absence of an effect was attributed to low coverage and use of household latrines [11,12]. The WASH for WORMS (W4W) RCT in Timor-Leste aimed to determine the additional benefit of an integrated community-based WASH and deworming intervention on STH infections when compared to deworming alone [13]. Surprisingly, also here no benefit arose from the WASH intervention with all the STH reduction arising from the PCT [14]. As such, in the context of calls for integration and scale-up of WASH and PCT experimental evidence for the benefit of WASH is limited. Mathematical modelling may shed some light on what WASH interventions can be expected to achieve in different settings and at different time scales.

In this paper we predict the impact of WASH interventions on STH in different epidemiological contexts with and without PCT, using a newly developed WASH extension of the established individual-based WORMSIM modelling framework for transmission and control of helminths [15]. We use the model to first explain the findings from the W4W study (i.e. no noticeable impact of latrines in context of community-wide PCT), then predict the short and long-term impact of different WASH modalities and the relative importance of uptake (proportion of population that takes up intervention) and effectiveness (impact of interventions on individuals that take up interventions). Finally, we investigate the added value of WASH to current control strategies for STH, in particular for sustaining achieved gains in the long run.

## Methods

### WORMSIM model

WORMSIM is a generalised individual-based modelling framework for transmission and control of helminth infections in humans, including soil-transmitted helminths [15], and is based on earlier individual-based models for onchocerciasis, schistosomiasis, and lymphatic filariasis [16–18]. WORMSIM predictions for STH have been previously validated against field data on trends in hookworm and *A*. *lumbricoides* infection levels before and during PCT [15,19]. Here we provide a high-level overview of the model; technical details can be found in S1 Text. A zip archive with the WORMSIM programme is provided in S1 File.

#### General model structure

WORMSIM explicitly simulates the life histories of individual humans and the individual worms living within humans. Simulated humans are exposed and contribute to a central reservoir of infection in the environment. Humans contribute infective material to the reservoir if female worms within them produce infective material (i.e. fertilized eggs), which is only possible after a period of pre-patency (maturation in the human host) and when at least one male worm is also present in the same host. Egg production by adult female worms is governed by negative density-dependence, which means that the egg output per female worm declines as the number of female worms in a host increases. The degree of parasite aggregation within the human population is governed by the level of inter-individual variation in exposure to the central reservoir of infection (by age, sex, and a random individual factor). Further, the model explicitly simulates individual host participation to PCT, accounting for age patterns and different degrees of systematic non-participation. Last, the model provides output in terms of predicted infection levels as observed by parasitological tests such as the Kato-Katz faecal slide method. For the current study, we expanded WORMSIM with model concepts for WASH interventions described below.

#### Environmental contamination and acquisition of infection

To explain the new model concepts for WASH interventions, we first describe how the model simulates the flow of infective material from hosts to the environmental reservoir and vice versa, which it does so in monthly time steps. Simulated humans containing reproductive adult worms contribute infective material to the environmental reservoir of infection. The amount of infective material contributed by a single simulated individual *i* in month *t* depends on age, sex, and a random personal factor as follows (details in S1 File):

$${\mathrm{contribution}}_{i,t}={\left[\mathrm{faecal}\;\mathrm{egg}\;\mathrm{output}\right]}_{i,t}*{\left[\mathrm{age}\;\mathrm{factor}\right]}_{i,t}*{\left[\mathrm{sex}\;\mathrm{factor}\right]}_{i}*{\left[\mathrm{random}\;\mathrm{personal}\;\mathrm{factor}\right]}_{i}$$

As a result of the contributions of all simulated individuals, infective material accumulates in the environmental reservoir over time. At the same time, during each time step of one month existing infective material in the environmental reservoir is assumed to decay at a user-defined exponential decay rate corresponding to the average lifespan of eggs or larvae in the environment (details in Supplemental File S1). Simulated individuals acquire new worm infections by exposure to the reservoir of infection, where the average force of infection (*FOI*_average,t_) on the human population in month *t* is equal to the amount of infective material in the reservoir in month *t* multiplied by parameter *ζ*, which can be tuned to reproduce some desired level of infection in the population. The *FOI*_*i*,*t*_ acting on a single simulated individual *i* during month *t* again depends on age, sex, and a random personal factor as follows: *FOI*_*i*,*t*_ = *FOI*_average,t_ * [age factor]_*i*,*t*_ * [sex factor]_*i*_ * [random personal factor]_*i*_. We assume that each individual’s random personal factor for exposure is equal to that for contribution, as these are most likely highly correlated through an individual’s behaviour (e.g. persons who practice more open defaecation are likely also more exposed to contaminated soil).

#### Model concepts for WASH

For modelling purposes, we define two WASH modalities: 1) “hygiene” interventions that reduce individuals’ exposure to infection (e.g. hand washing and shoe wearing), and 2) “sanitation” interventions that reduce individuals’ contribution to the environmental reservoir of infection (e.g. latrine use). The definition of these two modalities is not meant to exclude the “water” in “WASH”; we consider water to be of potential importance for either modality depending on what the water is needed for, e.g. to wash hands (hygiene modality) or to flush latrines (sanitation modality). We further define the impact of WASH in terms of uptake and effectiveness. Here, uptake represents the proportion of the population that takes up the WASH intervention, and effectiveness is defined at the individual level, i.e. the average reduction in exposure or contribution to transmission over time given that an individual takes up the intervention to some degree (but not necessarily benefiting from its maximum potential due to e.g. irregular or improper use).

The impact of hygiene interventions is defined by the term 1−(*α*_*H*_ * *β*_*H*,*i*,*t*_), which we multiply with the *FOI*_*i*,*t*_ acting on an individual in month *t*. Here, *β*_*H*,*i*,*t*_ is either zero or one representing whether or not individual *i* takes up the intervention in month *t*. Individual uptake *β*_*H*,*i*,*t*_ is determined by the user-defined overall population-level uptake of the intervention (a fraction between zero and one) in month *t* and an individual’s WASH participation index, which is a life-long random number between zero and one drawn from a uniform distribution: *β*_*H*,*i*,*t*_ = 1 if the individual’s participation index is smaller than or equal to the overall population-level uptake, and *β*_*H*,*i*,*t*_ = 0 otherwise. As a result, an individual’s uptake is considered constant over time if the population-level uptake is constant over time. Small-scale temporal (e.g. daily) variation in the actual uptake of the intervention is captured by parameter *α*_*H*_ (further referred to as “effectiveness”), which represents the average reduction in *FOI*_*i*,*t*_ over time in individuals who take up the intervention, given their compliance and the quality of the intervention. As such, values of *α*_*H*_ close to one (100% reduction) are probably unrealistic. Effectiveness *α*_*H*_ is further assumed to be the same for all individuals who take up the intervention. Different types or combinations of hygiene interventions (e.g. hand washing and/or shoe wearing) are only distinguished in terms of their uptake and effectiveness.

Similar to the above, the impact of sanitation interventions (subscript *S* instead of *H*) is represented by the term 1−(*α*_*S*_ * *β*_*S*,*i*,*t*_), which we multiply with each individual’s contribution to the environmental reservoir. Here, individual uptake *β*_*S*,*i*,*t*_ of sanitation interventions is based on the same individual participation index that feeds into individual uptake of hygiene interventions *β*_*H*,*i*,*t*_. This means that in case of simultaneously implemented sanitation and hygiene interventions, uptake of the two modalities is perfectly correlated within individuals (as long as population-level uptake of the two modalities is the same). Effectiveness parameter *α*_*S*_ represents the average reduction in individuals’ contribution to the reservoir over time.

Uptake of both hygiene and sanitation interventions are independent of individuals’ exposure or contribution to transmission. Further, we assume in the current study that uptake is independent of age because of community-wide implementation of WASH interventions. We further assume that uptake is identical to use and that frequency of use, compliance, and the effect of the intervention when actually using it are all captured by the effectiveness parameter (i.e. one minus uptake is the fraction of people who never use the WASH intervention). So by e.g. 70% uptake and 95% effectiveness we mean that the WASH intervention reduces the contribution and/or exposure to the environmental reservoir by 95% for 70% of people in the community.

### Simulations

The impact of PCT using ALB was simulated as in recent studies with WORMSIM [15,19]: i.e. assuming the drug kills 99% of *A*. *lumbricoides*, 95% of hookworms, and 60% of *T*. *trichiura* (unless specified otherwise). These percentages were based on faecal egg reduction rates (ERR) observed in a set of multi-country studies [20,21]. The frequency of PCT, age range of the target population, coverage of the target population, and pre-control transmission conditions were varied in different simulations.

Based on the self-reported level of latrine uptake in the W4W trial [14], we use a 70% population-level uptake of WASH interventions as a default value for our simulations, and specify an alternative hypothetical scenario with very high uptake of 95%. Because we do not know to what extent WASH interventions exactly reduce individuals’ contribution and/or exposure to the environmental reservoir, we take a qualitative approach and define two levels of effectiveness (70% and 95% reduction in individuals that take up intervention) such that we can compare the relative importance of uptake and effectiveness for the impact of WASH interventions on STH transmission. Explorative simulations with 100% uptake and effectiveness (which we do not consider realistic levels) showed little additional impact of WASH in the context of PCT compared to 95% uptake and effectiveness.

Because WORMSIM is a stochastic individual-based model, its predictions vary to some extent with repeated simulations when using the same input parameters (i.e. representing the potential courses of history in a probabilistic fashion). To predict the expected impact of interventions on STH transmission, we ran 100 repeated simulation for each scenario under consideration and took the mean of repeated simulations. To predict the probability of interruption of transmission, we ran 1000 repeated simulations and assessed what fraction of the simulations resulted in zero worm prevalence 50 years after stopping PCT.

## Results

**Fig 1:**
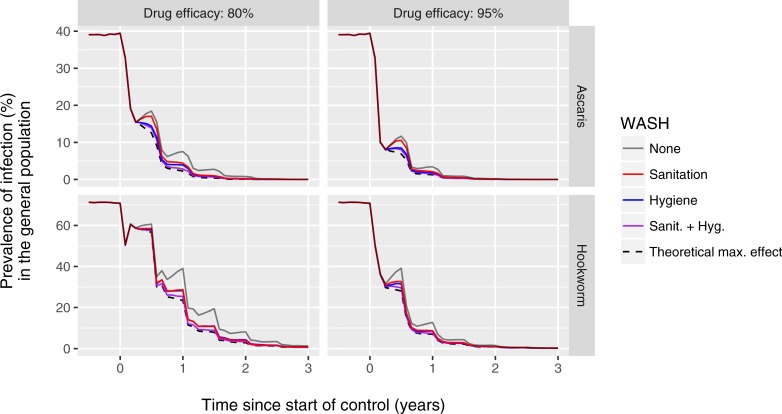
Model-predicted impact of community-wide WASH on prevalence of infection in the context of semi-annual community-wide preventive chemotherapy (PCT). The figure represents a setting highly endemic for *A*. *lumbricoides* and hookworm (rows of panels) where semi-annual community-wide deworming with is implemented at 80% population coverage. Drug treatment is assumed to kill either 95% or 80% of worms in treated individuals (columns of panels). Predicted prevalence of infection (y-axis) is based a single Kato-Katz faecal slide per individual. WASH interventions are assumed to be implemented at 70% uptake and 95% effectiveness. The dashed black line represents a theoretical scenario where sanitation and hygiene are perfectly implemented and taken up, reducing both exposure and contribution to transmission to zero for all individuals.

Fig 1 illustrates the predicted impact of 70% uptake of latrines on infection levels in the context of a community-wide deworming programme implemented at 80% coverage (as in the W4W trial area) in a setting with high pre-control prevalence of *A*. *lumbricoides* and hookworm infection (very few *T*. *trichuria* eggs were detected in the W4W trial area). In general, prevalence of infection, as detected by a single Kato-Katz faecal slide per person, declined steeply within two years of deworming (grey line). The additional impact of latrine use was very small (red line), even when assuming an effectiveness of 95%. This pattern was qualitatively similar when assuming normal or relatively low drug efficacy (95% vs. 80% of worms killed per treatment in treated individuals), or when using a hypothetical tests that can perfectly detect the density of adult female worms in a host or presence of at least one worm pair (S1 Fig), confirming the observation of little impact of latrine use in the context of community-wide PCT in the W4W trial [14].

Community-wide hygiene interventions or combined sanitation and hygiene intervention implemented at 70% uptake and 95% effectiveness were predicted to have little noticeable additional impact in the context of community-wide PCT (blue and purple lines in Fig 1), even when they are implemented and taken up perfectly such that no new infections can occur between PCT rounds (dashed black line). In contrast, the model predicts that the impact of the same community-wide WASH interventions would have been more readily detectable in the context of school-based deworming, which is logical as school-based deworming allows for the existence of a larger reservoir of infection in untreated individuals, leaving more potential impact for additional interventions (S2 Fig).

**Fig 2:**
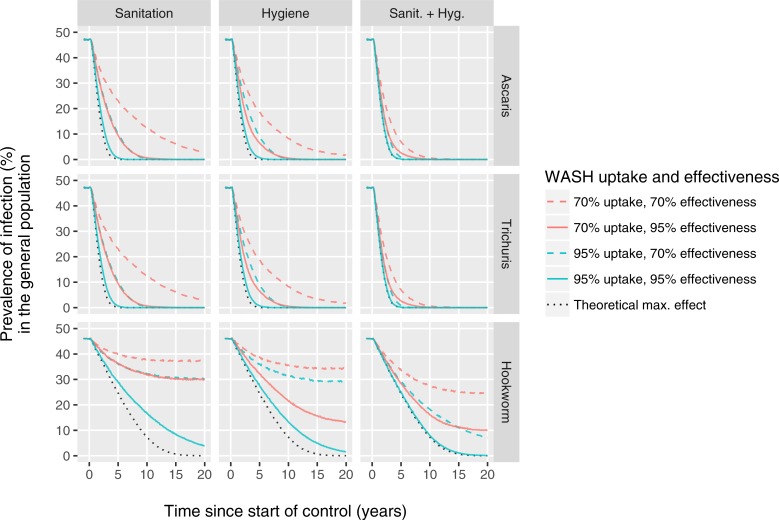
Model-predicted long-term impact of sanitation and hygiene interventions on prevalence of soil-transmitted helminth infection in absence of PCT. Columns of panels refer to different WASH modalities and rows represent the three major soil-transmitted helminth species. Sanitation and hygiene interventions are assumed to reduce the contributions and exposure to the environmental reservoir of infection of individuals who take up the intervention, respectively. Coloured lines indicate different levels of uptake. Different levels of effectiveness (reduction of contribution and/or exposure to the environmental reservoir for individuals that take up the intervention) are indicated by solid vs. dashed lines. The red solid dashed line is based on the same assumptions about WASH uptake and effectiveness used for Fig 1. The dotted black line represents a theoretical scenario where the WASH modality (of that column of panels) is perfectly implemented and taken up, reducing exposure and/or contribution to transmission by 100% for all individuals. Predicted infection prevalences (y-axis) represent results from a single Kato-Katz slide. See S3 Fig for similar figures for a wider range of levels of uptake and effectiveness.

Fig 2 shows the predicted impact of WASH interventions in a context without PCT. For the sake of comparing the impact on different species, we calibrated transmission for each species such that the model produced the same pre-control prevalence of infection of about 45%-50% (based on single Kato-Katz slide). For short-lived worms like *A*. *lumbricoides* and *T*. *trichiura*, both sanitation and hygiene can have a significant impact on infection levels within just two to five years. However, for hookworms the impact of sanitation and hygiene develops much more slowly (about four times slower).

To compare the relative importance of uptake and effectiveness for different WASH modalities, we plot the predicted impact of 70% uptake and 95% effectiveness (green dashed lines in Fig 2) vs. 95% uptake and 70% effectiveness (red solid lines). The impact of sanitation interventions (left column of panels) depends about equally on uptake and effectiveness: the green dashed and solid red lines are identical. In contrast, the impact of hygiene interventions (middle column of panels) depends more on effectiveness than on uptake: 70% uptake combined with 95% effectiveness has a higher impact than 95% uptake combined with only 70% effectiveness, especially for hookworm infections. This is a direct result of how hygiene interventions change the distribution of worms across hosts: if the worm burden is gradually reduced by 95% in 70% of people, there is reasonable chance that egg production will eventually stop in many of them. However, when worm burdens are reduced by only 70% in 95% of people, it is more likely that many people will still have at least one mated female worm producing eggs. Last, the impact of combined sanitation and hygiene interventions depends most on uptake (red solid line descends lower (eventually) than dashed green line), which is a direct consequence of our assumption that the two modalities are taken up by exactly the same individuals, creating a maximum synergistic effect in those who take up the interventions. See S3 Fig for similar figures as Fig 2, but for a wider range of levels of uptake and effectiveness.

**Fig 3:**
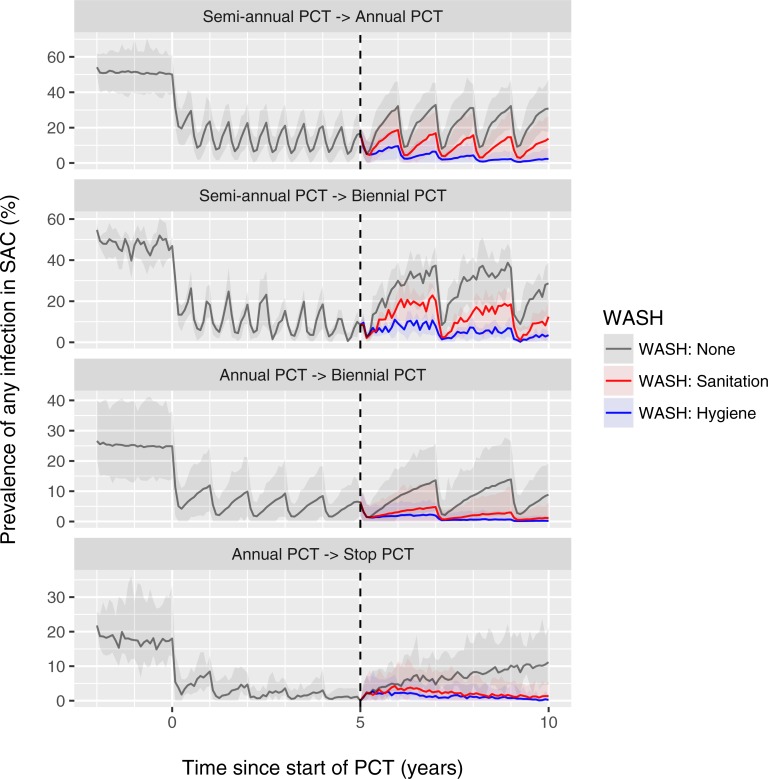
Model-predicted additional impact of WASH on hookworm prevalence in settings where school-based is scaled down or stopped. Lines represent the average of repeated simulations with random pre-control transmission conditions that followed a specific path through a decision tree for scaling down or stopping PCT as follows. Shaded areas represent the 2.5^th^ and 97.5^th^ percentiles of the stochastic variation in repeated simulations. For every single simulation, PCT was initiated at semi-annual or annual frequency (90% coverage of SAC) if pre-control infection prevalence in SAC was >50% or between 20% and 50%, respectively; at midline (after 5 years of PCT, dashed vertical line) the PCT frequency was scaled down from semi-annual to annual if prevalence of infection in SAC was between 10% and 20%, and was scaled down to biennial (from either annual or semi-annual) if the prevalence of infection was between 1% and 10%. PCT was stopped if the midline prevalence of infection in SAC was <1%. WASH interventions were assumed start at midline and to be taken up by 70% of the population and to result in a 95% reduction in participating individuals’ contribution (sanitation) or exposure (hygiene) to the environmental reservoir of infection. Pre-control endemicity differs between the panels because the different paths through the decision tree are more likely in some transmission conditions than others (e.g. the decision to stop PCT only comes up in situations with relatively favourable (low) pre-control endemicity). Depicted average trends are the net effect of potential interruption of transmission in some simulations and bounce-back in others. Similar figures are available for other STH species and settings where PCT is implemented community-wide (age 2 and above) at 75% coverage (S4 Fig).

Next, we investigate the potential impact of different WASH modalities in context of the current WHO guidelines for PCT, i.e. annual PCT with ALB or MEB targeted at children for areas with pre-control prevalence of STH infection of 20%-50% in SAC (as measured by single Kato-Katz slide) and semi-annual PCT for areas with pre-control prevalence >50%. The goal of this strategy is to reduce the prevalence of moderate-to-heavy intensity of infection to <1% in children and groups at high risk of morbidity such as women of childbearing age [4–6]. Although the operational goal is to achieve 75% PCT coverage of risk groups at the national level, we assume here that when implemented, school-based PCT covers 90% of the children in a community. Fig 3 shows the impact of these guidelines for those areas where school-based PCT is implemented but is scaled down or stopped after five years because of favourable results (<1%: stop PCT; 1–10%: bi-annual PCT; 10–20%: annual PCT), in absence and presence of different WASH modalities. Both sanitation and hygiene interventions (red and blue lines) were predicted to markedly reduce the bounce-back of infection levels after scaling down and to greatly prevent recrudescence of infection after stopping PCT altogether. Of note is that the impact of hygiene interventions is larger than that of sanitation interventions given the same level of uptake and effectiveness.

We performed a similar comparison for *A*. *lumbricoides* and *T*. *trichuris*, as well as areas where PCT is implemented community-wide, i.e. targeting the population of age 2 and above (S4 Fig). We conservatively assume that community-wide PCT is only able to achieve 75% coverage of the target population because of absence and/or refusal to participate. Because of the higher overall impact of community-wide treatment (despite lower coverage of target population), the decision to stop PCT after five years is taken in a greater proportion of the simulations, and even in situations with pre-control infection prevalence >50% in SAC (if the same decision criteria for stopping PCT are used as for school-based PCT). Given that the repercussions of stopping PCT too early are higher in areas with high pre-control infection levels (i.e. high transmission potential), WASH is even more important to prevent bounce-back of infection levels in areas with community-wide treatment.

**Fig 4:**
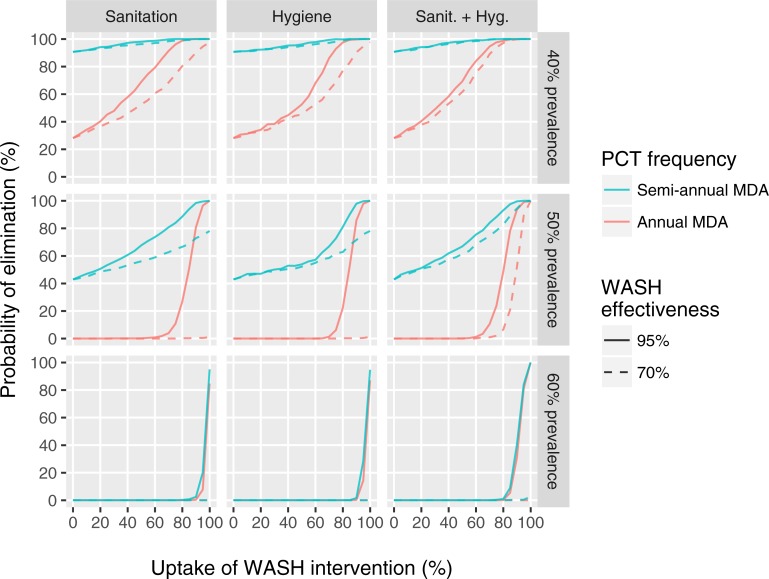
Effect of uptake and effectiveness of different WASH modalities on the probability of elimination of hookworm infection after five years of community-wide PCT. Rows of panels represent settings with different pre-control hookworm infection prevalence in the general population. For all three settings, we simulated five years of either annual (red lines) or semi-annual (green lines) community-wide PCT with ALB, implemented at 90% coverage. After five years, PCT is stopped and one of three types of WASH interventions (columns) is simulated, which reduce participating individuals’ contribution (sanitation) and/or exposure (hygiene) to the environmental reservoir of infection by 70% or 95% (effectiveness), represented by solid and dashed lines, respectively. Uptake of WASH interventions (x-axis) is defined as the (random) proportion of people who take up the intervention. Uptake of sanitation and hygiene measures in the combined WASH intervention (third column) is assumed to be perfectly correlated within individuals. Elimination is defined as absence of any worms 50 years after stopping PCT in the simulated population of about 400 individuals.

Last, Fig 4 illustrates the impact of uptake and effectiveness of different WASH modalities on probability of elimination hookworm infection after five years of PCT, where elimination is defined as zero worm prevalence 50 years after stopping PCT. The steep association between probability of elimination and WASH uptake suggests that uptake is a more important driver of elimination that WASH effectiveness, which is in contrast to Fig 2, where the impact of hygiene interventions on prevalence of infection is mostly driven by effectiveness of the intervention. This is logical as interruption of transmission is more difficult if a larger sub-group of individuals systematically does not take up interventions. Exceptions are situations where 70% effectiveness is not enough to achieve elimination because PCT has not driven down infection levels low enough (first and second panel of second row and all panels in bottom row). These patterns are similar for *A*. *lumbricoides* in areas with school-based deworming (S5 Fig; community-wide PCT quickly wipes out *A*. *lumbricoides*) and for *T*. *trichiura* in areas with community-wide deworming (S6 Fig; school-based PCT is unlikely to interrupt *T*. *trichiura* transmission), although achieving elimination with high probability requires generally lower levels of uptake in these contexts (given the same duration of PCT).

## Discussion

In this modelling study we confirm the finding from the W4W study that latrine use has little observable impact in the context of semi-annual community-wide deworming [14]. This is due the strong and quick impact of deworming masking the slower, more long-term impact of WASH, which can still be substantial. We further show that the impact of WASH interventions on STH transmission highly depends on the worm species, WASH modality, and pre-control endemicity (higher uptake is required for elimination in high prevalence settings). Also, the impact of WASH on STH infection levels is greatly diminished and slower for lower levels of uptake and effectiveness, which likely explains the lack of effect in the Indian Total Sanitation Campaign, as previously suggested [11,12]. Last, we show that WASH is particularly important to maintain gains when scaling down or stopping PCT, and when that happens, high uptake of WASH will be relatively more important than high effectiveness.

An important limitation of the modelling presented here is that the model quantifications for uptake and effectiveness of WASH in this study were largely hypothetical and not based on direct observations, except for the 70% figure of self-reported latrine use in the W4W trial. We initially did try to fit the uptake and effectiveness parameters in the model to the W4W trial to reproduce the observed infection levels over time, but this was complicated due to difficulties in interpretation of data on the impact of WASH because of potential counter-intended effects (e.g. latrines may actually act as sites of high exposure to infection) and the difficulties of quantifying self-reported human behaviour in general. Likewise, the use of a relatively new diagnostic tool (quantitative polymerase chain reaction [22]) for detection of infection posed difficulties in getting the model to exactly reproduce the data. We therefore chose to perform a more qualitative modelling study to disentangle the effects of different WASH modalities, to better understand potential interactions between WASH and PCT, and to help explain the patterns observed in the field, realising that not all hygiene and sanitation interventions are necessarily equal in terms of uptake or effectiveness in reality. If the uptake and/or effectiveness of WASH interventions are lower in field settings than assumed in our simulations, the impact of such interventions will be even more difficult to measure than predicted here. If on the other hand uptake and/or effectiveness of WASH interventions are higher in reality than assumed here, it will still be difficult to measure their impact in the context of community-wide deworming due to the large impact of PCT on infection levels, even when using a highly accurate test as was done in the W4W trial.

To better demonstrate the benefit and importance of WASH and better inform mathematical models with plausible estimates of the effectiveness and uptake of WASH interventions, we recommend that the impact of WASH interventions is evaluated in settings where expected reinfection rates are considerable, i.e. settings where PCT is implemented only annually (as opposed to semi-annually) and/or targeted only at certain risk groups (e.g. school-age children, as opposed to community-wide PCT) (see also Box 1). It might even be useful to evaluate the impact of WASH in areas where pre-control STH prevalence is so low (<20%) that PCT need not be started according to WHO guidelines [6], although it could be challenging to measure intervention impact in context of low infection prevalence due to relatively low statistical power. Although reinfection rates are also high in highly endemic areas, this does not guarantee that an effect of WASH, if any, can be detected during community-wide PCT, as was the case for the W4W study [14]. Although challenging, collection of individual-level longitudinal data on both WASH-related behaviour and infection levels would provide a wealth of information for mathematical models. Further, in addition to WASH uptake and effectiveness, the epidemiological impact of WASH in trials or observational studies will also depend on the longevity of eggs and or larvae in the local environment, which may vary between studies depending on environmental factors such as temperature, humidity, etc. Therefore, analyses of such data with mathematical models will require assumptions or actual data on the survival of eggs and larvae; here we assume “default” average life expectancies based on literature, but with more data, the model could be improved to represent specific local situations.

**Box 1. Recommendations for future WASH trials**

- Impact of WASH interventions is best demonstrated based on longitudinal individual-level data from settings with considerable reinfection rates, e.g. settings where PCT programmes is school-based (as opposed to community-based) and implemented annually (vs. semi-annually).
- Studies aiming to inform models on uptake and effectiveness of WASH should also assess the survival of eggs/larvae in the environment.
- Future WASH trials should better disentangle interventions that are intended to reduce exposure vs. contribution to the environmental reservoir and should provide detailed reports on access to and use of each modality.
- Future WASH trials should focus on the long-term impact.
- Future WASH trials should carefully monitor and correlate uptake of WASH interventions with pre-control infection status.
- Design of future WASH trials can significantly benefit from exploratory mathematical modelling of the expected effects.

We show that the effects of WASH on STH infection levels is of a lesser magnitude and occurs at a slower rate for hookworm than for *A*. *lumbricoides* and *T*. *trichuris*, which can be directly explained by the relatively short average lifespan of adult *A*. *lumbricoides* and *T*. *trichuris* worms (about one year vs. three years for hookworms) [23–27]. Due to their shorter lifespan, and despite the relatively long average lifespan of eggs in the environment (months vs. weeks for hookworm), *A*. *lumbricoides* and *T*. *trichuris* infection levels in the human population change more quickly in response to changes in the environmental reservoir or exposure to the reservoir than for hookworm. A second contributing factor is that egg productivity per adult female hookworm [28] is relatively low compared to *A*. *lumbricoides* [24] or *T*. *trichiura* [29], which means that for a given species-specific prevalence of egg-positivity, humans need to harbour relatively more (mated) female hookworms, and as a result, it takes longer for natural attrition (or deworming) to reduce the worm population in size to the point that infected individual stops excreting eggs.

In a recent meta-analysis of observational and intervention studies, Freeman *et al* [3] found that the association between sanitation and *A*. *lumbricoides* infection is stronger than for the other STHs (odds ratio of 0.77, compared to 1.0 for whipworm and 0.94 for hookworm). The authors hypothesised that this pattern may be explained by the potent effect of ALB on *A*. *lumbricoides*, reasoning that sanitation itself is unlikely to reduce infection over a short period of time and only helps reduce reinfection. We however show here that 1) potent PCT interventions mask the impact of WASH, and that 2) with sufficient uptake and effectiveness of sanitation in the absence of PCT, *A*. *lumbricoides* and *T*. *trichiura* infection prevalence decline much faster (substantially so within a year) than hookworm prevalence, which in itself may already explain the observation of a difference between hookworm and roundworm in terms of strength of the association with sanitation. Moreover, drug efficacy is high for both *A*. *lumbricoides* (ERR 99% and 98% for ALB and MEB, respectively) and hookworm (ERR 96% and 81%) [20,21]. Therefore, we expect that the association with sanitation is stronger for *A*. *lumbricoides* than for hookworm because of shorter worm lifespan, as well as the fact that the impact of school-based deworming is higher for *A*. *lumbricoides* than for hookworm because most of the worms reside in children, in contrast to hookworms, for which densities are highest in (often untreated) adults [19,30]. Still, the difference in strength of association with sanitation between *A*. *lumbricoides* and *T*. *trichiura* may well indeed be partly attributable to low drug efficacy against *T*. *trichiura* (ERR 65% and 63%).

Previous WHO guidelines included the option to scale down or stop PCT if sufficient impact has been achieved after five to six years [4]. These options are no longer mentioned in a recent update of the guidelines [6], but might still be considered in some places, either in the context of an STH control programme, or when stopping community-wide PCT against lymphatic filariasis, which includes the distribution of ALB (combined with ivermectin outside Africa, which is also highly efficacious against *T*. *trichiura* [31]). A recent modelling study already highlighted the importance of setting up contingency mechanisms when stopping PCT against STH [32]. Here, we add further evidence about how WASH can strongly reduce the risk and speed of bounce-back of infection levels after stopping PCT, which supports the notion that integration of WASH and PCT policy is important for long-term sustained STH control.

We present the first attempt at modelling the impact of WASH on STH transmission. Our approach comes with three important limiting assumptions. The first is that that the assumed effectiveness of WASH interventions is the same for everybody who takes up WASH. The actual effect of WASH in terms of reduction in contribution and exposure to the environmental reservoir may however vary between individuals due to inter-individual variation in frequency and quality of execution or use of the intervention. Second, we assume that uptake of hygiene and sanitation is perfectly correlated in individuals, allowing for a maximum synergistic effect between the two WASH modalities. If in reality individuals have a particular preference for one of the two modalities (e.g. one subgroup prefers hygiene interventions and another prefers sanitation), then such synergistic effects become less prominent. Third, we assume that uptake and effectiveness of WASH is independent of an individual’s infection status before start of interventions. In light of the possibility that uptake and effectiveness of WASH is higher in individuals with lower infection status (better socio-economic status and/or more time, knowledge, and self-efficacy to actually use WASH), the impact of WASH in field settings may be lower than currently predicted by our model. These three assumptions are subject of future studies, which require further extensions of the WASH model concepts. Future modelling will also cover the impact of school-based WASH in contrast to community-based WASH, which will require detailed modelling of multiple environmental reservoirs in school and household areas.

In conclusion, we show that the impact of WASH interventions on STH transmission highly depends on the worm species, WASH modality, and uptake and effectiveness of the intervention. Also, the impact of WASH is difficult to measure in the context of ongoing deworming programmes. Still, we show a clear added benefit of WASH to sustain the gains made by PCT in the long term, such that PCT may be scaled down or even stopped altogether. All of the above support the notion that WASH and PCT policy should be integrated.

## Supporting information

S1 Text Technical model description. (DOCX)

S1 Fig Model-predicted impact of community-wide WASH on intensity of infection (page 1) and prevalence of worm pairs (page 2) in the context of semi-annual community-wide deworming. The figure represents a setting highly endemic for *A*. *lumbricoides* and hookworm (rows of panels) where semi-annual community-wide deworming is implemented at 80% population coverage. Drug treatment is assumed to kill either 95% or 80% of worms in treated individuals (columns of panels). Predicted prevalence of infection (y-axis) is based a hypothetical test that perfectly detects the density of adult female worms in a host. WASH interventions, if any, are assumed to be implemented at 70% uptake and 95% effectiveness. The dashed black line represents a theoretical scenario where WASH is implemented perfectly such that transmission stops from the first PCT round onwards.
(PDF)

S2 Fig Model-predicted impact of community-wide WASH on soil-transmitted helminths in the general population (odd pages) and school-aged children (even pages) in the context of semi-annual school-based deworming. The figure represents a setting highly endemic for *A*. *lumbricoides* and hookworm (rows of panels) where semi-annual school-based deworming is implemented at 90% coverage of school-age children (SAC). Drug treatment is assumed to kill either 95% or 80% of worms in treated individuals (columns of panels). WASH interventions, if any, are assumed to be implemented at 70% uptake and 95% effectiveness. The dashed black line represents a theoretical scenario where WASH is implemented perfectly such that transmission stops from the first PCT round onwards. In this theoretical scenario, the small inter-treatment rebounds in infection levels in SAC are due to previously untreated pre-school age children entering the SAC age group.
(PDF)

S3 Fig Model-predicted long-term impact of sanitation and hygiene interventions on prevalence of soil-transmitted helminth infection in absence of PCT. Columns of panels refer to different WASH modalities and rows represent the three major soil-transmitted helminth species. Sanitation and hygiene interventions are assumed to reduce the contributions and exposure to the environmental reservoir of infection of individuals who take up the intervention, respectively. Coloured lines indicate different levels of uptake. Different levels of effectiveness (reduction of contribution and/or exposure to the environmental reservoir for individuals that take up the intervention) are indicated by solid vs. dashed vs. dashed-dotted lines. The dotted black line represents a theoretical scenario where the WASH modality (of that column of panels) is perfectly implemented and taken up, reducing exposure and/or contribution to transmission by 100% for all individuals. Predicted infection prevalences (y-axis) represent results from a single Kato-Katz slide.
(PDF)

S4 Fig Model-predicted additional impact of WASH in settings where school-based or community-based PCT is scaled down or stopped. Lines represent the average of repeated simulations with random pre-control transmission conditions that followed a specific path through a decision tree for scaling down or stopping PCT as follows. Shaded areas represent the 2.5^th^ and 97.5^th^ percentiles of the stochastic variation in repeated simulations. For every single simulation, PCT was initiated at semi-annual or annual frequency (90% coverage of SAC or 75% of population of age 2 and above) if pre-control infection prevalence in SAC was >50% or between 20% and 50%, respectively; at midline (after 5 years of PCT, dashed vertical line) the PCT frequency was scaled down from semi-annual to annual if prevalence of infection in SAC was between 10% and 20%, and was scaled down to biennial (from either annual or semi-annual) if the prevalence of infection was between 1% and 10%. PCT was stopped if the midline prevalence of infection in SAC was <1%. WASH interventions were assumed start at midline and to be taken up by 70% of the population and to result in a 95% reduction in participating individuals’ contribution (sanitation) or exposure (hygiene) to the environmental reservoir of infection. Pre-control endemicity differs between the panels because the different paths through the decision tree are more likely in some transmission conditions than others (e.g. the decision to stop PCT only comes up in situations with relatively favourable (low) pre-control endemicity). Depicted average trends are the net effect of potential interruption of transmission in some simulations and bounce-back in others.
(PDF)

S5 Fig Effect of uptake and effectiveness of different WASH modalities on the probability of elimination of *A*. *lumbricoides* infection after five years of school-based PCT (community-based PCT quickly interrupts *A*. *lumbricoides* transmission). Rows of panels represent settings with different pre-control *A*. *lumbricoides* infection prevalence in the general population. For all three settings, we simulated five years of either annual (red lines) or semi-annual (green lines) school-based PCT with ALB, implemented at 90% coverage. After five years, PCT is stopped and one of three types of WASH interventions (columns) is simulated, which reduce participating individuals’ contribution (sanitation) and/or exposure (hygiene) to the environmental reservoir of infection by 70% or 95% (effectiveness), represented by solid and dashed lines, respectively. Uptake of WASH interventions (x-axis) is defined as the (random) proportion of people who take up the intervention. Uptake of sanitation and hygiene measures in the combined WASH intervention (third column) is assumed to be perfectly correlated within individuals. Elimination is defined as absence of any worms 50 years after stopping PCT in the simulated population of about 400 individuals. Lines for semi-annual and annual (red and green) are very close because neither strategy leads to a situation close to elimination after only 5 years of school-based PCT.
(PDF)

S6 Fig Effect of uptake and effectiveness of different WASH modalities on the probability of elimination of *T*. *trichiura* infection after five years of community-based PCT (school-based PCT is often not sufficient to interrupt *T*. *trichiura* transmission). Rows of panels represent settings with different pre-control *T*. *trichiura* infection prevalence in the general population. For all three settings, we simulated five years of either annual (red lines) or semi-annual (green lines) community-wide PCT with ALB, implemented at 90% coverage. After five years, PCT is stopped and one of three types of WASH interventions (columns) is simulated, which reduce participating individuals’ contribution (sanitation) and/or exposure (hygiene) to the environmental reservoir of infection by 70% or 95% (effectiveness), represented by solid and dashed lines, respectively. Uptake of WASH interventions (x-axis) is defined as the (random) proportion of people who take up the intervention. Uptake of sanitation and hygiene measures in the combined WASH intervention (third column) is assumed to be perfectly correlated within individuals. Elimination is defined as absence of any worms 50 years after stopping PCT in the simulated population of about 400 individuals.
(PDF)

S1 File ZIP Archive with WORMSIM version 2.58Ap27 and three example input files for *A*. *lumbricoides*, *T*. *trichiura*, and hookworm *spp*. (ZIP)
