# Supplementary material for: Predicted short and long-term impact of deworming and water, hygiene, and sanitation on transmission of soil-transmitted helminths
Source: PLoS Negl Trop Dis. 2018 Dec 6;12(12):e0006758. doi: 10.1371/journal.pntd.0006758 (PMC6283645; doi:10.1371/journal.pntd.0006758)
Supplement: S1 Text — (DOCX) [file pntd.0006758.s001.docx]

# S1 Supplemental Text: Formal description of WORMSIM

Luc E. Coffeng^ab^*, Susana Vaz Nery^b^, Darren J. Gray^b^, Roel Bakker^a^, Sake J. de Vlas^a^, Archie C.A. Clements^b^

^a^ Department of Public Health, Erasmus MC, University Medical Center Rotterdam, Rotterdam, The Netherlands

^b^ Research School of Population Health, College of Medicine, Biology and Environment, The Australian National University, Canberra, Australian Capital Territory, Australia

* Corresponding author: [l.coffeng@erasmusmc.nl](mailto:l.coffeng@erasmusmc.nl), +31 10 70 38464

## Table of contents

Table of contents 2

1. This document 3

2. Introduction 3

3. Formal description of WORMSIM (v2.58Ap27) 4

Human demography 4

Transmission of infection 5

Uptake of hygiene and sanitation interventions 9

Mass treatment coverage and compliance 9

Parasitological effects of treatment 11

Vector control 12

Surveys 12

Simulation warm-up 12

References 19

## This document

This document provides a description of the WORMSIM model structure and default parameter quantification as used in the simulations for the paper *Predicted short and long-term impact of deworming and WASH on transmission of soil-transmitted helminths* by Coffeng *et al* (PLoS Negl Trop Dis 2018).

## Introduction

WORMSIM is a generalised framework for modelling transmission and control of helminth infections in humans. It is based on previous individual-based models for onchocerciasis (ONCHOSIM), schistosomiasis (SCHISTOSIM), and lymphatic filariasis (LYMFASIM) [1–3], and has been recently extended to facilitate modelling of soil-transmitted helminths [4]. WORSIM simulates the life histories of individual helminths and their transmission from person to person mediated by a “cloud” that can be taken to represent a vector population or an environmental reservoir. In addition, WORMSIM can be used to evaluate the effects of different control strategies, such as vector control and chemotherapy. WORMSIM combines two simulation techniques; *stochastic microsimulation* is used to calculate the life events of individual persons and their inhabitant parasites, while the dynamics of infective material in the cloud (i.e. the vector population or environmental reservoir) is simulated *deterministically*.

The version of WORMSIM used in this study (v2.58Ap27) is based on the C++ code of ONCHOSIM, but has been redesigned and extended using object-oriented principles and has been programmed in Java. Individual people and mature worms are modelled as distinct objects. WORMSIM is event-driven, which means that time progresses as a result of events (although monthly events are used for most processes). The main advantages of the implementation in Java are improved code quality and therefore easier maintenance and extension. Model input parameters are specified in a structured XML-file, which is automatically validated using an XML Schema before the start of a set of simulations.

In section 3, we describe the general structure of the modelling framework. In footnotes we highlight details and alternative options that are not evident from the mathematical descriptions. The WORMSIM framework is very flexible in that it allows the user to choose probability distributions for stochastic processes and functional relationships for deterministic processes, and to change the associated parameter values. Table A and Table B at the end of this document provide an overview available distributions and functions. Table C provides an overview of the parameter values used in this particular study.

## Formal description of WORMSIM (v2.58Ap27)

### Human demography

The human population dynamics is governed by birth and death processes. We define *F(a)* as the probability to survive to age *a* (Table 3). The cumulative survival for intermediate ages is obtained by linear interpolation.

The expected number of births (per year) at a given moment *t* is given by:

| $R_{b}\left( t \right)=\sum_{a=1}^{n_{a}} N_{f}\left( a,t \right)\cdot r_{b}\left( a \right)$ | (1) |
| --- | --- |

with:

$N_{f}\left( a,t \right)$ number of women in age group *a* at time *t*

$r_{b}\left( a \right)$ annual birth rate in age-group *a* (Table 3).

$n_{a}$ number of age-groups considered.

Each month, $R_{b}\left( t \right)$ is adapted according to the number of women and their age-distribution.

Once every year, the total number of human individuals is checked; if the total number is larger than a user-defined value, a fraction (also user-defined) is randomly removed from the simulation.

The population distribution resulting from the aforementioned parameters is illustrated in Figure A, and closely follows the age distribution in Sub-Saharan Africa as estimated by the UN Population Division for the year 2000 (Figure A) [5].

Figure A. Population demography simulated in WORMSIM in absence of excess mortality due to disease (bars), compared to the 2000 population for Sub-Saharan Africa (diamonds; UN Population Division, World Population Prospects: The 2012 Revision).

### Transmission of infection

In this section we describe how WORMSIM simulates a full transmission cycle in absence of interventions, which involves human exposure to infection and acquisition of worms, dynamics of infection within humans, contribution of infective material to the cloud, and within-cloud dynamics of infective material. The cloud represents a vector population (e.g. for onchocerciasis or lymphatic filariasis) or an environmental reservoir of infection (e.g. for soil-transmitted helminths). Individual human hosts are *exposed* to the infective material in the cloud at varying rates, given their age, sex, and personal factors. Vice versa, individual hosts *contribute* infective material (larvae or eggs) to the cloud, the amount depending on the number and reproductive statues of worms in the individual, as well as an individual host’s contribution rate (depending on age, sex, and personal factors), and potential limited processes (e.g. density-dependent uptake of infective material by vectors). The amount of infective material in the cloud is updated in discrete monthly time steps.

#### Exposure to infection and acquisition of new worms

First, we define the overall force of infection $lr\left( t \right)$ acting on the human population in month *t* as a function of the current absolute amount of infective material in the cloud $\bar{lu}\left( t \right)$:

| $lr\left( t \right)=\bar{lu}\left( t \right)\cdot\zeta\cdot v$ | (2) |
| --- | --- |

Here, $\zeta$ (zeta) is a scalar representing the overall exposure rate, and *v* is the probability that an infective particle in the reservoir successfully develops into a parasite life stage that is capable of infecting a human host.^[[1]](#footnote-1)^

Next, we define the force of infection acting upon individual *i* of age *a* and sex *s* as:

| ${foi}_{i}(t)=lr(t)\cdot\frac{{Ex}_{i}}{\sum_{i=1}^{N\left( t \right)} {Ex}_{i}}\cdot\left( 1-\alpha_{H}\cdot\beta_{H,i,t} \right)$ | (3) |
| --- | --- |

Here, ${Ex}_{i}$ is the relative exposure of an individual, taking into account age *a* and sex *s*, as well as personal factors:

| ${Ex}_{i}=Exa\left( a_{i},s_{i} \right)\cdot{Exi}_{i}$ | (4) |
| --- | --- |

with:

$Exa\left( a_{i},s_{i} \right)$ Relative exposure of person with age *a* and sex *s*, defined as a linearly interpolated function of user-defined exposure rates for a finite set of ages (for each sex).

${Exi}_{i}$ Exposure index of person *i*, which captures personal factors related to e.g. behaviour and occupation. ${Exi}_{i}$ is assumed to follow a gamma distribution with mean 1.0 and shape and rate (or 1/scale) equal to $\alpha_{Exi}$. The exposure index of a person remains constant throughout lifetime.^[[2]](#footnote-2)^

The term $\left( 1-\alpha_{H}\cdot\beta_{H,i,t} \right)$ represents the impact of hygiene-related interventions^[[3]](#footnote-3)^ that individual *i* participates in at time *t*, with:

$\alpha_{H}$ Reduction in force of infection when taking up the hygiene intervention (assumed to be the same for all individuals who participate).

$\beta_{H,i,t}$ Indicator (0 or 1) for whether individual *i* takes up the hygiene intervention at time *t*.

Finally, a person *i* is assumed to become infected at time *t*, according to a Poisson process with rate equal to ${foi}_{i}(t)\cdot sr\cdot{Imm}_{i}(t)$. Here, success ratio *sr* is a constant representing the probability that an inoculated infective particle will develop into a macroparasite.^a^ Finally, ${Imm}_{i}(t)$ represents the impact of the host’s immune response at time *t* on incoming infections [6]:

| ${Imm}_{i}\left( t \right)=1-\alpha_{imm}\cdot{imm}_{i}\cdot W_{cum,i}\left( t \right)$ | (5) |
| --- | --- |
| $W_{cum,i}\left( t \right)=W_{i}\left( t \right)+\beta_{Imm}\cdot W_{cum,i}\left( t-1 \right)$ |  |
|  |  |

Here, $\alpha_{imm}$ is the effect of immunity and ${imm}_{i}$ is an individual host’s capacity to elicit an immune response, drawn from a positive bounded probability distribution with mean one (e.g. a gamma distribution with equal shape and rate (1/scale) parameters). $W_{cum,i}\left( t \right)$ is the cumulatively experienced worm burden of host *i* in month *t*, $W_{i}\left( t \right)$ is the worm burden of host *i* in month *t*, and $\beta_{Imm}$ represents the immunological memory span ($\beta_{Imm}=e^{-\ln\left( 2 \right)/\lambda_{imm}}$, where $\lambda_{imm}$ is the desired half-life (in months) of the immunological response; vice vera $\lambda_{imm}={-\ln\left( 2 \right)}/{\ln\left( \beta_{Imm} \right)}$).

#### Within-host dynamics of infection

For convenience, in this section we drop the subscript *i* for individual humans. The lifespan of male and female parasites within human hosts is a random variable: $Tl\sim\text{Weibull}\left( \mu_{Tl},\alpha_{Tl} \right)$, with mean $\mu_{Tl}$ years and shape $\alpha_{Tl}$.^[[4]](#footnote-4)^ Once parasites come of age (i.e. when they pass the prepatent age $pp$), female worms can start producing larvae or eggs, and males can inseminate female worms. The reproductive capacity $r\left( a,t \right)$ of a patent female worm of age *a* at time *t* is calculated as follows (in absence of drug effects):

| $r\left( a,t \right)=R(a-pp)\cdot m(t)$ | (6) |
| --- | --- |

with:

$R(A)$ Potential reproductive capacity of a female worm, *A* years after reaching patency, defined as a linearly interpolated function of user-defined values for a finite set of ages.

$m(t)$ Mating factor at time *t*

To produce larvae or eggs, a female worm must be inseminated each reproductive cycle $rc$, defined in terms of months. If insemination took place less than $rc$ months ago, then $m\left( t \right)=1$. Otherwise, the probability of insemination or reinsemination $P_{ins}\left( t \right)$ in month *t* is given by:

| $P_{ins}\left( t \right)=\left\{ \begin{aligned} pot{\cdot W}_{m}\left( t \right)/W_{f}\left( t \right)\text{ if }\text{ }{pot\cdot W}_{m}\left( t \right)<W_{f}\left( t \right) \\ 1\text{ if }pot<0\text{ or otherwise} \end{aligned} \right.$ | (7) |
| --- | --- |

with:

$W\left( t \right)$ the number of male ($W_{m}$) or female ($W_{f}$) parasite in the human at time *t*

$pot$ the number of female worms that a male worm can inseminate per month^[[5]](#footnote-5)^

If no insemination takes place then $m\left( t \right)=0$ and the female worm has a new opportunity to be inseminated in the next month *t* + 1. If insemination occurs in month *t_i_* then $m\left( t \right)=1$ during *t_i_ ≤ t <t_i_ + rc*.

The density of larvae (e.g. per skin snip) or eggs (e.g. per gram faeces) $sl\left( t \right)$ in a host at time *t* is calculated by averaging the production of all female parasites over the past *Tm* months within that host:

| $sl\left( t \right)=O\left( el\left( t \right) \right)$ | (8) |
| --- | --- |
| $el\left( t \right)=\frac{1}{Tm}\sum_{j}^{n_{i}} d_{j}\sum_{x=1}^{Tm} r_{j}\left( a_{j}-x,t-x \right)$ | (9) |

with:

$el\left( t \right)$ the *effective parasite load* at time *t*. This intermediate variable describes the female parasite load obtained by weighting each worm according to the mf-productivity during the past *Tm* months.^[[6]](#footnote-6)^

$O\left( . \right)$ A function that returns the total amount of infective material produced by female parasites. For soil-transmitted helminths, we assume that $O\left( . \right)$ is the hyperbolic saturating function (see Table 2) of the number of female worms.^[[7]](#footnote-7)^

$d_{j}$ *dispersal factor* of female parasite *j*. This is a random variable (mean 1.0) drawn for every “newborn” worm, and accounts for differences in the contribution of female worms to the density at the standard site of the body where samples are taken or vectors bite.

$Tm$ (fixed) lifespan of larvae or eggs within the host in terms of months.

$n_{i}$ number of parasites alive during at least one of the months *t-1,…,t-Tm*.

#### Host contribution of infective material to the cloud

Given the density of larvae or eggs ${sl}_{i}\left( t \right)$ in all $N\left( t \right)$ host in month *t*, the total amount of infective material that is contributed to the cloud by the host is defined as

| ${\bar{lu}\left( t \right)}_{in}=\sum_{i=1}^{N\left( t \right)} Mbr\left( t \right)\cdot rbr\cdot U\left( {sl}_{i}(t) \right)\cdot{Co}_{i}\cdot\left( 1-\alpha_{S}\cdot\beta_{S,i,t} \right)$ | (10) |
| --- | --- |

Here, $Mbr\left( t \right)$ is the average contribution rate in month *t* (*monthly biting rate* for filarial infections), allowing the user to define a seasonal pattern (in absence of vector control). The relative biting rate *rbr* is used to scale this seasonal pattern to some desired level.^a^ The function $U\left( . \right)$ returns the amount of infective material taken up by the cloud given the density of eggs or larvae ${sl}_{i}\left( t \right)$ in a host, possibly in a density dependent manner to represent e.g. limited vectorial capacity to transmit infection.^[[8]](#footnote-8)^ The term ${Co}_{i}$ is the relative contribution of an individual, given age, sex, and personal factors:

| ${Co}_{i}=Coa(a_{i},s_{i})\cdot{Coi}_{i}$ | (11) |
| --- | --- |

with:

$Coa\left( a_{i},s_{i} \right)$ Relative contribution of person with age *a* and sex *s*, defined as a linearly interpolated function of user-defined exposure rates for a finite set of ages (for each sex).

${Coi}_{i}$ Contribution index of person *i*, which captures personal factors related to e.g. behaviour and occupation. ${Coi}_{i}$ is assumed to follow a gamma distribution with mean 1.0 and shape and rate (or 1/scale) equal to $\alpha_{Coi}$. The contribute index of a person remains constant throughout lifetime. In WORMSIM default assumption is that ${Coi}_{i}={Exi}_{i}$, unless separate distributions are defined.

The term $\left( 1-\alpha_{S}\cdot\beta_{S,i,t} \right)$ represents the impact of sanitation-related interventions^[[9]](#footnote-9)^ that individual *i* participates in at time *t*, with:

$\alpha_{S}$ Reduction in an individual’s environmental contamination rate when taking up the sanitation intervention (assumed to be the same for all individuals who participate).

$\beta_{S,i,t}$ Indicator (0 or 1) for whether individual *i* takes up the sanitation intervention at time *t*.

#### Dynamics of infective material in the cloud

For the dynamics of infective material in the cloud we define a deterministic, discrete model:

| $\bar{lu}\left( t \right)={\bar{lu}\left( t \right)}_{in}+\psi\cdot\bar{lu}\left( t-1 \right)$ | (12) |
| --- | --- |

Each month, new infective material ${\bar{lu}\left( t \right)}_{in}$ is added to the cloud, and a fixed proportion $\psi$ of the infective material from the previous month is carried over, assuming exponential survival of infective material. The average life span of infective material in the cloud is then defined as ${-1}/{\ln(\psi)}$ months. To simulate filarial transmission, we set $\psi=0$, such that the cloud represents a vector population in which larvae survive for much shorter than a month. To simulate STH infections, we set $0<\psi<1$, such that the cloud represents an environmental reservoir of infection in which infective material survives for a non-negligible time.

### Uptake of hygiene and sanitation interventions

As briefly explained in the previous section, in WORMSIM we define hygiene and sanitation interventions in terms of individual uptake $\beta_{X,i,t}$ ($X\in\left( S,H \right)$) and individual-level impact $\alpha_{X}$ in case of uptake. Individual uptake $\beta_{X,i,t}$ is determined by the user-defined overall population-level uptake of the intervention at time *t* (a fraction between zero and one) and an individual’s participation index for hygiene and sanitation interventions, which is a life-long random number between zero and one drawn from a uniform distribution: $\beta_{X,i,t}=1$ if the individual’s participation index is smaller than or equal to the overall population-level uptake, and $\beta_{X,i,t}=0$ otherwise. As a result, an individual’s uptake is considered constant over time if the population-level uptake is constant over time. Small-scale temporal (e.g. daily) variation in the actual uptake of the intervention is captured by parameter $\alpha_{X}$, which can be taken to represent the average reduction in ${foi}_{i}$ over time in individuals that take up the intervention. As such, values of $\alpha_{X}$ close to one are probably unrealistic. Further, $\alpha_{X}$ is assumed to be the same for all individuals who take up the intervention.

Uptake of both hygiene and sanitation interventions are independent of individuals’ exposure or contribution to transmission. Further, although it is possible in WORMSIM to vary population-level uptake by age, we assume that uptake is independent of age because of community-wide implementation of WASH interventions.

### Mass treatment coverage and compliance

The primary characteristic of a certain ivermectin mass treatment *w* is the coverage $C_{w}$ (fraction of the population treated). However, a difficulty in calculating individual chances of participation is that there are several exclusion criteria for the drug. Moreover, compliance to treatment differs from person to person. Exclusion criteria can be either permanent (chronic illness) or transient (e.g. related to age or pregnancy). We define the population that potentially participates as the total population *minus* a fraction $f_{c}$ that never participates in mass drug administration. The coverage among the potentially participating population ${C'}_{w}$ is now given by:

| ${C'}_{w}={C_{w}}/\left( 1-f_{c} \right)$ | (15) |
| --- | --- |

Here, ${C'}_{w}$ cannot be larger be than one (i.e. is capped off at one).

To capture transient contra-indications and other age- and sex-related factors for participation in mass treatment, we define the age- and sex-specific relative compliance $c_{r}\left( k,s \right)$ (Table 3). Note that in $c_{r}\left( k,s \right)$ only the *ratio* between the values for the different groups is relevant.

Now, the coverage $c\left( k,s,w \right)$ in each of the age- and sex-groups (among people that potentially participate) at treatment round *w* is calculated as:

| $c\left( k,s,w \right)=\frac{c_{r}\left( k,s \right)\cdot N\left( w \right)}{\sum_{s=1}^{2} \sum_{k=1}^{n_{a}} c_{r}\left( k,s \right)\cdot N\left( k,s,w \right)}\cdot{C'}_{w}$ | (16) |
| --- | --- |

with:

$N\left( k,s,w \right)$

Number of individuals eligible to treatment in age-group *k* and sex *s* at treatment round *w*.

$N\left( w \right)$ Total number of eligible individuals at treatment round *w*.

Finally, the probability to participate in treatment round *w* for an person *i* of age-group *k* and sex *s* is given by:

| ${Ptr}_{i,w}={{co}_{i}}^{\frac{1-c\left( k,s,w \right)}{c\left( k,s,w \right)}}$ | (17) |
| --- | --- |

with:

${co}_{i}$ Personal compliance index. This is considered as a lifelong property and is generated by a uniform distribution on [0,1]

Note that for all *k* and *s* the average value of ${Ptr}_{i,w}$ equals$c\left( k,s,w \right)$. Now, in WORMSIM we define 3 coverage models. In model 0, the probability to be treated is as given in equation (17). In model 1, the probability is equal to $c\left( k,s,w \right)$ and the compliance index ${co}_{i}$ is ignored. The simplest model is model 2 in which the treatment probability simply equals *C’_w_*. All models take account of a fraction $f_{c}$ of permanently excluded persons. Figure B illustrates the impact of different assumptions about compliance patterns on the proportion of the population that has never been treated after a certain number of treatment rounds.

Figure B. Relation between compliance patterns and proportion of population that has never been treated. For simplicity, here we assume that compliance is independent of age and sex. Random compliance (solid line) means that eligible individuals participate completely at random (compliance model 1 or 2 in WORMSIM, depending on whether age and sex-patterns are required). Systematic compliance (dotted line) means that an individual either always participates (if eligible) or never (compliance model 1 or 2 in WORMSIM, combined with a fraction of excluded people equal to one minus the target coverage). The mixed compliance pattern (dashed line) means that some individuals are systematically more likely to participate than others (but everyone will participate at some point; compliance model 0 in WORMSIM).

### Parasitological effects of treatment

In WORMSIM, drug treatment affects parasites in three main ways. First, a drug may instantly kill a proportion of larvae or eggs present in a host. This proportion is either fixed or a randomly drawn from a user-defined probability distribution for each host and treatment.

Second, a drug may instantly kill pre-patent and adult worms with probability $m_{i}$ in host *i*. A worm *j* dies when a random variate $u_{j}$ on [0,1] (redrawn for every new treatment) is smaller than or equal to $m_{i}$.

Third, a drug may temporarily and/or permanently (and cumulatively) reduce the reproductive capacity of female worm by a proportion $d_{i}$ in host *i*. In case of a temporary effect, the reproductive capacity will restore within a period ${Tr}_{i}$ to its maximum value (in case of any concomitant permanent reductions, reproductive capacity will regenerate to the new, permanently reduced maximum value. The second and third effect are jointly defined as follows:

| $m_{i}=v_{i}m_{0}$ |  | (18) |
| --- | --- | --- |
| $d_{i}=v_{i}d_{0}$ |  |  |
| ${Tr}_{i}=v_{i}{Tr}_{0}$ |  |  |
| $r_{j}\left( a_{j},t \right)=r_{j}^{0}\left( a_{j},t \right)\cdot\left( 1-d_{i} \right)\cdot\left( \frac{\tau}{{Tr}_{i}} \right)^{s}$, | if $u_{j} >m_{i}$, $d_{i} <1$, and $t<{Tr}_{i}$ |  |
| $r_{j}\left( a_{j},t \right)=r_{j}^{0}\left( a_{j},t \right)\cdot\left( 1-d_{i} \right)$ | if $u_{j} >m_{i}$, $d_{i} <1$, and $t\geq{Tr}_{i}$ |  |
| $r_{j}\left( t \right)=0$ | otherwise |  |

with:

*v_i_* Relative effectiveness of treatment in person *i*. For every separate treatment and person, a new value is drawn for *v_i_* from a user-defined distribution (i.e. the relative effectiveness applies to all worms in a person during a specific treatment).

$m_{0}$ Average fraction of prepatent and adult parasites killed.

$d_{0}$ Average permanent (unrecoverable) reduction in female reproductive capacity.

${Tr}_{0}$ Average duration until full recovery from temporary effects on female reproductive capacity.

$r_{j}\left( a_{j},t \right)$

Reproductive capacity of female worm *j* in month *t*, $\tau$ months after the last treatment.

$r_{j}^{0}\left( a_{j},t \right)$

Reproductive capacity of female worm *j* had person *i* not been treated at the last round, $\tau$ months ago.

$s$ Shape parameter of the recovery function.

In addition to this, we explicitly consider that some persons (a user-defined random fraction of the treated population) do not at all react to the drug during a certain treatment due to malabsorption (e.g. due to vomiting or diarrhoea).

### Vector control

Vector control is modelled as a reduction of the monthly biting rates during a given period of time. A period of vector control^[[10]](#footnote-10)^ is specified as the year + month of the beginning of the strategy and the year + month of the end of a strategy. If a certain month during a period of *d* days larvicides have been applied, then the reduction in $Mbr\left( t \right)$ in that month equals *d*/30 x 100%.

### Surveys

During the simulation, user-defined surveys will take place. During a survey, for all simulated individuals the actual number of male and female worms is recorded, and a diagnostic test is simulated to detect infective material (larvae, eggs). For the diagnostic test, the expected amount of infective material per sample (e.g. microfilariae per skin snip, or eggs per gram faeces) for an individual is given by ${sl}_{i}(t)$.

The actual number of infective particles (microfilariae, eggs, etc.) in the sample is assumed to follow a discrete distribution like a Poisson or negative binomial distribution, with mean equal to ${sl}_{i}(t)$ (see Table 1 for available distributions).^[[11]](#footnote-11)^ At each epidemiological survey a user-defined number of samples are taken from all simulated persons, for which the results are averaged (per simulated person). The results of such a survey are post-processed to arrive at age and sex-specific prevalences and intensities of infection.

### Simulation warm-up

In general, before starting simulation of interventions in ONCHOSIM, a 200-year warm-up period is simulated, such as to allow the human and worm population to establish equilibrium levels, given the parameters for average fly biting rate and inter-individual variation in exposure to infection. At the start of the warm-up period, an artificial force of infection is simulated for a user-defined number of years, allowing worms to establish themselves in the human population (here: 5 worms per person per year for 5 years). After the 200 warm-up years, the simulated infection levels are no longer correlated with the initial conditions at the start of the warm-up period.

Table A. Probability distributions available in WORMSIM. Reference numbers (rightmost column) are used in the WORMSIM input file to define probability distributions for stochastic processes. Within WORMSIM, stochastic processes are pre-defined to follow either a continuous or discrete distribution, so each type has its own list of reference numbers.

| **Probability distribution** | **Parameters in input file** | **Domain** | **Probability density function** | **Reference number** |
| --- | --- | --- | --- | --- |
| *Continuous distributions* | | | | |
| Constant (real) | $\mu$ | $\mu$ | $f\left( x \right)=1$ | 0 |
| Uniform | $\mu$, $p_{1}$ | ${(p}_{1},2\mu-p_{1})$ | $f\left( x \right)={(2\mu)}^{-1}$ | 1 |
| Exponential | $\mu$ | $\mathbb{R}^{+}$ | $f\left( x \right)={\mu^{-1}e}^{-x/\mu}$ | 2 |
| Weibull | $\mu$, $p_{1}$ | $\mathbb{R}^{+}$ | $f\left( x \right)={{{\alpha\beta}^{-\alpha}x^{\alpha-1}e}^{-\left( x/\beta\right)}}^{\alpha}$,  where $\alpha=p_{1}$ and $\beta=\mu/{\Gamma\left( 1+1/\alpha\right)}$ | 3 |
| Gamma | $\mu$, $p_{1}$ | $\mathbb{R}^{+}$ | $f\left( x \right)=\frac{x^{k-1}e^{-x/\theta}}{\Gamma\left( k \right)\theta^{k}}$,  where $k=p_{1}$ and $\theta=\mu/p_{1}$ | 4 |
| Beta (optionally scaled) | $\mu$, $p_{1}$, $p_{2}$ | $\left( 0,p_{2} \right)$ | $f\left( x_{1} \right)=\frac{\Gamma\left( \alpha+\beta\right)}{\Gamma\left( \alpha\right)\Gamma\left( \beta\right)}x_{trans}^{\alpha-1}\left( 1-x_{trans} \right)^{\beta-1}$,  where $x_{trans}=x/p_{2}$, $\alpha=p_{1}$, and $\beta=p_{1}\left( \frac{p_{2}}{\mu}-1 \right)$ | 5 |
| Normal | $\mu$, $p_{1}$ | $\mathbb{R}$ | $f\left( x \right)=\frac{1}{\sqrt{2\pi\sigma^{2}}}e^{-\left( x-\mu\right)^{2}/2\sigma^{2}}$,  where $\sigma=p_{1}$ | 6 |
| Log-normal | $\mu$, $p_{1}$ | $\mathbb{R}^{+}$ | $f\left( x \right)=\frac{1}{x\sqrt{2\pi\sigma_{ln}^{2}}}e^{-\left( \text{ln}\left( x \right)-\mu_{ln} \right)^{2}/2\sigma_{ln}^{2}}$,  where $\mu_{ln}=\ln\left( \frac{\mu^{2}}{\sqrt{p_{1}^{2}+\mu^{2}}} \right)$, and $\sigma_{ln}^{2}=\ln\left( \frac{p_{1}^{2}+\mu^{2}}{\mu^{2}} \right)$; i.e. $\mu$ and $p_{1}$ are defined on the positive real plane $\mathbb{R}^{+}$. | 7 |
| *Discrete distributions* | | | | |
| Constant (integer) | $\mu$ | $\left\Vert\mu\right\Vert$ | $f\left( x \right)=1$ | 0 |
| Bernoulli* | $\mu$ | $\mathbb{Z}_{1}$ | $f\left( x \right)=\mu^{x}\left( 1-\mu\right)^{1-x}$ | 1 |
| Uniform* | $\mu$ | $\mathbb{Z}_{j+1}$ | $f\left( x \right)=\frac{1}{j+1}$, where $j=\left\lfloor\left( 2m+0.5 \right) \right\rfloor$ and $\left\lfloor y \right\rfloor$ is the largest integer not larger than $y$ | 2 |
| Binomial* | $\mu$, $p_{1}$ | $\mathbb{Z}_{p_{1}}$ | $f\left( x \right)=\binom{n}{x}\mu^{x}\left( 1-\mu\right)^{n-x}$, where $n=p_{1}$ | 3 |
| Geometric* | $\mu$ | $\mathbb{Z}^{*}$ | $f\left( x \right)=\left( 1-p \right)^{x}p$, where $p=\frac{1}{\mu+1}$ | 4 |
| Poisson | $\mu$ | $\mathbb{Z}^{*}$ | $f\left( x \right)=\frac{e^{-\mu}\mu^{x}}{x!}$ | 5 |
| Negative binomial | $\mu$, $p_{1}$ | $\mathbb{Z}^{*}$ | $f\left( x \right)=\left( \frac{k}{k+\mu} \right)^{k}\frac{\Gamma\left( k+x \right)}{x!\Gamma\left( k \right)}\left( \frac{\mu}{k+\mu} \right)^{x}$, where $k=p_{1}$ | 6 |
| * This distribution was available in the original ONCHOSIM model, but is still due to be implemented in WORMSIM. The Bernoulli distribution is used in parts of the model, but is hardcoded in these cases. The geometric distribution can be simulated by means of a negative binomial distribution with $p_{1}=1$. In a future update of WORMSIM, all listed distributions will be (re-)implemented using the Apache Commons Math library. | | | | |

Table B. Functional relationships available in WORMSIM. Reference numbers (rightmost column) are used in the WORMSIM input file to define functional relationships for deterministic processes.

| **Functional relationship** | **Parameters** | **Formula** | **Parameter constraints and function characteristics** | **Reference number** |
| --- | --- | --- | --- | --- |
| Constant | $a$ | $f\left( x \right)= a$ | $a\geq0$ | 0 |
| Linear | $a$, $b$, $c$ | $f\left( x \right)=ax+b$ if $ax+b<c$,  $f\left( x \right)=c$ otherwise | $a\geq0$; $b\geq0$; if $c<0$, no maximum is considered | 1 |
| Hyperbolic saturating | $a$, $b$, $c$ | $f\left( x \right)=c\cdot\frac{ax}{1+ax/b}$ | $b\geq0$; $c\geq0$; $ba>0$; $f\left( 0 \right)=0$; $\lim_{x\to\infty} f(x)=b$; $f'\left( 0 \right)=a$ | 2 |
| Exponential saturating | $a$, $b$, $c$ | $f\left( x \right)=a\left( 1-e^{-bx} \right)\left( 1+e^{-cx} \right)$ | $a\geq0$; $b>0$; $c\geq0$; $f\left( 0 \right)=0$; $\lim_{x\to\infty} f(x)=a$; $f'\left( 0 \right)=2ab$ | 3 |
| Sigmoidal saturating | $a$, $b$, $c$ | $f\left( x \right)=a\left( 1-e^{-\left( bx \right)^{c}} \right)$ | $a\geq0$; $b>0$; $0.1<c<10$; $f\left( 0 \right)=0$; $\lim_{x\to\infty} f(x)=a$; $f'\left( 0 \right)=0$for $c>1$;  $f'\left( 0 \right)=ab$for $c=1$;  $f'\left( 0 \right)=\infty$for $c<1$ | 4 |
| Power function | $a$, $b$ | $f\left( x \right)=ax^{b}$ | $a\geq0$; $b\geq0$; $f\left( 0 \right)=0$; $\lim_{x\to\infty} f(x)=\infty$;  $f'\left( 0 \right)=0$for $b>1$;  $f'\left( 0 \right)=a$for $b=1$;  $f'\left( 0 \right)=\infty$for $b<1$ | 5 |

Table C. WORMSIM quantification used to simulate hookworm transmission. Given that WORMSIM is a general modelling framework that covers both STH and filariasis, certain parameters do not apply to hookworm transmission but are listed anyway for the sake of completeness (indicated where applicable). Further, within the groups of model parameters for transmission and surveys, certain parameters are strongly correlated as indicated by “*not identified*”. Where this is the case, set all but one parameter to arbitrary values, and then used the one parameter to tune the model, as indicated by “*used as main parameter…*”.

| **Parameter** | **Value** | **Source** |
| --- | --- | --- |
| **Human demography** | | |
| Cumulative survival ($F\left( a \right)$ by age | | [5] |
| Africa | 1.000, 0.804, 0.772, 0.760, 0.740, 0.686, 0.509, and 0.000 at ages 0, 5, 10, 15, 20, 30, 50, and 90, respectively. |  |
| Timor Leste | 1.000, 0.950, 0.936, 0.879, 0.751, and 0.000 at ages 0, 1, 5, 40, 60, and 90, respectively. |  |
| Fertility rate per woman ($R\left( a \right)$) by age | | [5] |
| Africa | 0.000, 0.109, 0.300, and 0.000 for age groups 0–14, 15–29, 30–49, and 50+, respectively. |  |
| Timor Leste | 0.000, 0.0658, 0.2707, 0.3040, 0.2762, 0.2181, 0.1157, 0.0555, and 0.000 for age groups 0-15, 15-20, 20-25, 25-30, 30-35, 35-40, 40-45, 45-50, and 50-90, respectively. |  |
| Population trimming | 10% if population size exceeds 500. | Assumption |
| **Transmission of infection** | | |
| *General transmission parameters* |  |  |
| Relative biting rate (*rbr*) | $rbr=1$; applies only to filarial transmission; not identified. |  |
| Overall exposure rate of human hosts to central reservoir of infection ($\zeta$) | Tuned to produce some desired overall level of infection in the simulated population. | Tuned |
| Seasonal variation in contribution to reservoir (*mbr*) | Stable throughout the year. | Assumption |
| Transmission probability (*v*) | $v=1$; not identified. |  |
| Success ratio (*sr*) | $sr=1$; not identified. |  |
| Zoophily (*z*) | $z=0$; not applicable. |  |
| *Individual relative exposure to cloud* |  |  |
| Variation in by age and sex (*Exa*) |  |  |
| Hookworm | Linearly increasing from 0 to 1 between ages 0–10 and stable thereafter; no difference between males and females. | [4] |
| Ascaris and trichuris | Linearly increasing from 0.33 to 1 between ages 0–3, linearly declining from 1 to 0.33 between ages 15–20, and stable at 0.33 from age 20 onwards; no difference between males and femals. | [7] |
| Variation due to personal factors (fixed through life) given age and sex ($\alpha_{Exi}$) | Tuned to produce some desired distribution of intensity of infection in the simulated population, if data is available. If not, default values based on analysis of external field data are used. |  |
| Hookworm | $\alpha_{Exi}=0.35$ | [8] |
| Ascaris | $\alpha_{Exi}=0.8$ | [9] |
| Trichuris | $\alpha_{Exi}=0.35$ | [10] |
| *Individual relative contribution to cloud* | | |
| Variation by age and sex (*Coa*) | Linearly increasing from 0 to 1 between ages 0–10 and stable thereafter; no difference between males and females; same for all worm species. | [4,7] |
| Variation due to personal factors (fixed through life) given age and sex ($\alpha_{Coi}$) | Assumed to be perfectly correlated with individual exposure to reservoir, given age and sex (i.e. $Coi=Exi$). | Assumption |
| *Host immunity to incoming infections* | |  |
| Average impact of host immunity ($\alpha_{Imm})$ | $\alpha_{Imm}=0$; i.e. no effect of immunity on incoming infections. | Assumption |
| Immunological memory ($\beta_{Imm})$ | Irrelevant given that $\alpha_{Imm}=0$. | Assumption |
| **Life history and productivity of the parasite in the human host** | | |
| Average worm lifespan (*Tl*) |  |  |
| Hookworm | 3 years | [11–13] |
| Ascaris | 1 year | [9,11–14] |
| Trichuris | 1 year | [11–13] |
| Variation in worm lifespan | Weibull distribution with shape 2; i.e. the mortality rate is zero at age zero and then increases linearly with age. | Assumption |
| Prepatent period (*pp*) |  |  |
| Hookworm | 7 weeks | [11,12,15,16] |
| Ascaris | 10 weeks | [11] |
| Trichuris | 10 weeks | [11] |
| Age-dependent reproductive capacity (*R(a)*) | *R(a)* = 1 for patent female worms of any age. | Assumption |
| Longevity of infective material within host (*Tm*) | 1 month; i.e. the minimum given that transmission is simulated in discrete time steps of one month. | Assumption |
| Mating cycle (*rc*) | 1 month; i.e. the minimum given that transmission is simulated in discrete time steps of one month. | Assumption |
| Male potential (*pot*) | 100 female worms. | Assumption |
| *Density-dependent female worm reproductive capacity* |  |  |
| Female worm fecundity | Density-dependent on total number of female worms in host, assuming hyperbolic saturation with inter-individual variation between individuals in the potential maximum total host output, as described by a gamma distribution with mean 1.0 and a shape and rate of 50 (95%-CI: 0.74–1.30). | [4] |
| Hookworm | $a_{o}=200$ epg per female worm, as previously reported based on association between number of expulsed adult female worms and egg counts based on Kato-Katz. The average maximum total host output is assumed to be 1500 epg (as measured by Kato-Katz), as previously assumed [4]. | [17] |
| Ascaris | $a_{o}=9750$ epg per female worm, and maximum total host output of 18,650 epg. These figures were estimated from pre-control data on number of expulsed adult female worms and egg counts based on a concentration and sedimentation technique using homogenised stools. | [9] |
| Trichuris | $a_{o}=370$ epg per female worm, and maximum total host output of 80,000 epg. | [10] |
| **Morbidity** | | |
| Disease threshold (*Elc*) | Not used. |  |
| Reduction in remaining life expectancy due to disease (*rl*) | Not used. |  |
|  |  |  |
| **Infection dynamics in the cloud** | | |
| Cloud uptake of infectious material ($U\left( . \right)$) | The identity function, meaning there is no density-dependence in uptake of infective material by the environmental reservoir. | Assumption |
| Monthly cumulative survival of infective material in the central reservoir ($\psi$) | General assumption: survival of infective material is exponential |  |
| Hookworm | $e^{\left( -\frac{52}{2}\cdot\frac{1}{12} \right)}=11.5\%$, given an average lifespan of two weeks (95%-CI: 0.05–7.38 weeks under assumption of exponential survival), based on the notion that average survival is a matter of “weeks” according to literature. | [15,16,18] |
| Ascaris | $e^{\left( -\frac{1}{1.5} \right)}=51.3\%$, given an average lifespan of 1.5 month, (95%-CI: 0.04–5.53 months under assumption of exponential survival) . | [12,13] |
| Trichuris | $e^{\left( -\frac{3}{2} \right)}=51.3\%$, given an average lifespan of 2/3 of a month, (95%-CI: 0.02–2.46 months under assumption of exponential survival) . | [12,13] |
| **Drug treatment** | | |
| Proportion of larvae or eggs cleared from host | 0% | Assumption |
| Duration of temporary reduction in female reproductive capacity (${Tr}_{0}$) | 0 months | Assumption |
| Permanent reduction in female worm reproductive capacity ($d_{0}$) | 0% | Assumption |
| Proportion of adult worms killed ($m_{0})$ |  |  |
| Hookworm | $m_{0}=0.95$ for albendazole and $m_{0}=0.80$ for mebendazole. | [19] |
| Ascaris | $m_{0}=0.99$ for albendazole and $m_{0}=0.98$ for mebendazole. | [19] |
| Trichuris | $m_{0}=0.60$ for albendazole, $m_{0}=0.60$ for mebendazole, and $m_{0}=0.95$ for albendazole + ivermectin. | [19,20] |
| Relative effectiveness (*v*) | *v* = 1 (constant, i.e. no additional variation). | Assumption |
| **Surveys** |  |  |
| Dispersal factor for worm contribution to measured density of infective material (*d*) | d = 1; constant, i.e. no additional variation; not identified. |  |
| Variability in measured host load of infective material (eggs per gram faeces) | Negative binomial distribution with mean $ss\left( t \right)$ and aggregation $k$. |  |
| Hookworm | $k=0.32$ | [21] |
| Ascaris | $k=0.4$ |  |
| Trichuris | $k=0.25$ |  |
| Cut-offs for no, light, moderate, and heavy infection |  | [22] |
| Hookworm | 1, 2000, and 4000 epg |  |
| Ascaris | 1, 5000, and 50,000 epg |  |
| Trichuris | 1, 1000, and 10,000 epg |  |

## References

1. Plaisier AP, van Oortmarssen GJ, Habbema JD, Remme J, Alley ES (1990) ONCHOSIM: a model and computer simulation program for the transmission and control of onchocerciasis. *Comput Methods Programs Biomed* **31**: 43–56.

2. de Vlas SJ, Van Oortmarssen GJ, Gryseels B, Polderman AM, Plaisier AP, et al. (1996) SCHISTOSIM: a microsimulation model for the epidemiology and control of schistosomiasis. *Am J Trop Med Hyg* **55**: 170–175.

3. Plaisier AP, Subramanian S, Das PK, Souza W, Lapa T, et al. (1998) The LYMFASIM simulation program for modeling lymphatic filariasis and its control. *Method Inf Med* **37**: 97–108.

4. Coffeng LE, Bakker R, Montresor A, de Vlas SJ (2015) Feasibility of controlling hookworm infection through preventive chemotherapy: a simulation study using the individual-based WORMSIM modelling framework. *Parasit Vectors* **8**: 541.

5. United Nations Department of Economic and Social Affairs Population Division (2013) World Population Prospects: The 2012 Revision, Volume I: Comprehensive Tables.

6. Anderson RM, May RM (1985) Herd immunity to helminth infection and implications for parasite control. *Nature* **315**: 493–496.

7. Coffeng LE, Truscott JE, Farrell SH, Turner HC, Sarkar R, et al. (2017) Comparison and validation of two mathematical models for the impact of mass drug administration on Ascaris lumbricoides and hookworm infection. *Epidemics* **18**: 38–47.

8. Sarkar R, Rose A, Mohan VR, Ajjampur SSR, Veluswamy V, et al. (2017) Study design and baseline results of an open-label cluster randomized community-intervention trial to assess the effectiveness of a modified mass deworming program in reducing hookworm infection in a tribal population in southern India. *Contemp Clin Trials Commun* **5**: 49–55.

9. Elkins DB, Haswell-Elkins M, Anderson RM (1986) The epidemiology and control of intestinal helminths in the Pulicat Lake region of Southern India. I. Study design and pre- and post-treatment observations on Ascaris lumbricoides infection. *Trans R Soc Trop Med Hyg* **80**: 774–792.

10. Farrell SH, Coffeng LE, Truscott JE, Werkman M, Toor J, et al. (2018) Investigating the Effectiveness of Current and Modified World Health Organization Guidelines for the Control of Soil-Transmitted Helminth Infections. *Clin Infect Dis* **66**: S253–S259.

11. Bethony J, Brooker S, Albonico M, Geiger SM, Loukas A, et al. (2006) Soil-transmitted helminth infections: ascariasis, trichuriasis, and hookworm. *Lancet* **367**: 1521–1532.

12. Anderson RM, Truscott J, Hollingsworth TD (2014) The coverage and frequency of mass drug administration required to eliminate persistent transmission of soil-transmitted helminths. *Philos Trans R Soc L B Biol Sci* **369**: 20130435.

13. Truscott JE, Hollingsworth TD, Brooker SJ, Anderson RM (2014) Can chemotherapy alone eliminate the transmission of soil transmitted helminths? *Parasit Vectors* **7**: 266.

14. Croll NA, Anderson RM, Gyorkos TW, Ghadirian E (1982) The population biology and control of Ascaris lumbricoides in a rural community in Iran. *Trans R Soc Trop Med Hyg* **76**: 187–197.

15. Hotez PJ, Brooker S, Bethony JM, Bottazzi ME, Loukas A, et al. (2004) Hookworm infection. *N Engl J Med* **351**: 799–807.

16. Brooker S, Bethony J, Hotez PJ (2004) Human Hookworm Infection in the 21st Century. *Adv Parasitol*. Vol. 58. pp. 197–288.

17. Anderson RM, Schad GA (1985) Hookworm burdens and faecal egg counts: an analysis of the biological basis of variation. *Trans R Soc Trop Med Hyg* **79**: 812–825.

18. Augustine DL (1923) Investigations on the control of hookworm disease. XVI. Length of life of hookworm larvae from the stools of different individuals. *Am J Epidemiol* **3**: 127–136.

19. Levecke B, Montresor A, Albonico M, Ame SM, Behnke JM, et al. (2014) Assessment of anthelmintic efficacy of mebendazole in school children in six countries where soil-transmitted helminths are endemic. *PLoS Negl Trop Dis* **8**: e3204.

20. Belizario VY, Amarillo ME, de Leon WU, de los Reyes AE, Bugayong MG, et al. (2003) A comparison of the efficacy of single doses of albendazole, ivermectin, and diethylcarbamazine alone or in combinations against Ascaris and Trichuris spp. *Bull World Heal Organ* **81**: 35–42.

21. Pullan RL, Kabatereine NB, Quinnell RJ, Brooker S (2010) Spatial and Genetic Epidemiology of Hookworm in a Rural Community in Uganda. *PLoS Negl Trop Dis* **4**: e713.

22. Albonico M, Bickle Q, Ramsan M, Montresor A, Savioli L, et al. (2003) Efficacy of mebendazole and levamisole alone or in combination against intestinal nematode infections after repeated targeted mebendazole treatment in Zanzibar. *Bull World Heal Organ* **81**: 343–352.

1. $\zeta$ is perfectly negatively correlated with transmission probability *v*, success ratio *sr*, relative biting rate *rbr*, and vector zoophily *z*. See also the section on contribution of infective material to reservoir. For filarial transmission, we set $\zeta=1$, quantify *v* based on vector biology, set *sr* to a constant value, and calibrate transmission with *rbr*. For STH, we set $v=rbr=sr=1$ , and calibrate transmission with $\zeta$, which has a more natural explanation in the STH context (exposure to the reservoir) than *rbr*. [↑](#footnote-ref-1)
2. If desired, other continuous probability functions can be chosen. [↑](#footnote-ref-2)
3. We choose to use the term “hygiene” here as the main paper is about soil-transmitted helminths. However, the same model concepts can be used to simulate the impact of e.g. bed net use on transmission of vector-born helminths. [↑](#footnote-ref-3)
4. For readers used to the other commonly used parameterization of the Weibull distribution in terms of shape *k* and scale λ, shape *k* is *α_Tl_* (as described in this document) and scale $\lambda={\mu_{Tl}}/{\Gamma\left( 1+1/{\alpha_{Tl}} \right)}$. [↑](#footnote-ref-4)
5. When the user specifies a negative value for *male potential*, female worms can produce larvae or eggs in the absence of male worms. [↑](#footnote-ref-5)
6. In a future update of WORMSIM, $el\left( t \right)$ will be defined as the weighted sum of $r_{j}\left( a_{j}-x,t-x \right)$ over the last $Tm$ months (instead of the average). [↑](#footnote-ref-6)
7. Alternatively, a linear or other functional relationship between *el* and *sl* can be defined. Saturating functions should not be used when $Tm>1$, as this will cause partial saturation of female worm productivity in month *t*, given the output in months $t-1$ through $t-Tm$. This will be alleviated in a future version of WORMSIM by setting $sl\left( t \right)=\sum_{x=1}^{Tm} O\left( \sum_{j}^{n_{i}} r_{j}\left( a_{j}-x,t-x \right) \right)$. [↑](#footnote-ref-7)
8. For filarial infection, $U\left( . \right)$ typically is a density-dependent function of ${sl}_{i}(t)$ to represent limited vectorial capacity to transmit infection, whereas for STH, we take $U\left( . \right)$ to be the identity function. [↑](#footnote-ref-8)
9. We choose to use the term “sanitation” here as the main paper is about soil-transmitted helminths. However, the same model concepts can be used to simulate the impact of e.g. bed net use on transmission of vector-born helminths. [↑](#footnote-ref-9)
10. Multiple periods of vector control can be specified, each with its own effectiveness. [↑](#footnote-ref-10)
11. For filariasis transmission, we typically assume that sampling error is Poisson distributed. For faecal egg counts in STH infection, we assume that sampling error is negative binomial, while setting $d_{j}$=1. For hookworm, we assume that aggregation parameter $k=0.4$, based on an analysis of field data [21], kindly provided by Simon Brooker. [↑](#footnote-ref-11)
