# Supplementary material for: Predicted short and long-term impact of deworming and water, hygiene, and sanitation on transmission of soil-transmitted helminths
Source: PLoS Negl Trop Dis. 2018 Dec 6;12(12):e0006758. doi: 10.1371/journal.pntd.0006758 (PMC6283645; doi:10.1371/journal.pntd.0006758)
Supplement: S1 Fig — The figure represents a setting highly endemic for A. lumbricoides and hookworm (rows of panels) where semi-annual community-wide deworming is implemented at 80% population coverage. Drug treatment is assumed to kill either 95% or 80% of worms in treated individuals (columns of panels). Predicted prevalence of infection (y-axis) is based a hypothetical test that perfectly detects the density of adult female worms in a host. WASH interventions, if any, are assumed to be implemented at 70% uptake and 95% effectiveness. The dashed black line represents a theoretical scenario where WASH is implemented perfectly such that transmission stops from the first PCT round onwards. (PDF) [file pntd.0006758.s002.pdf]

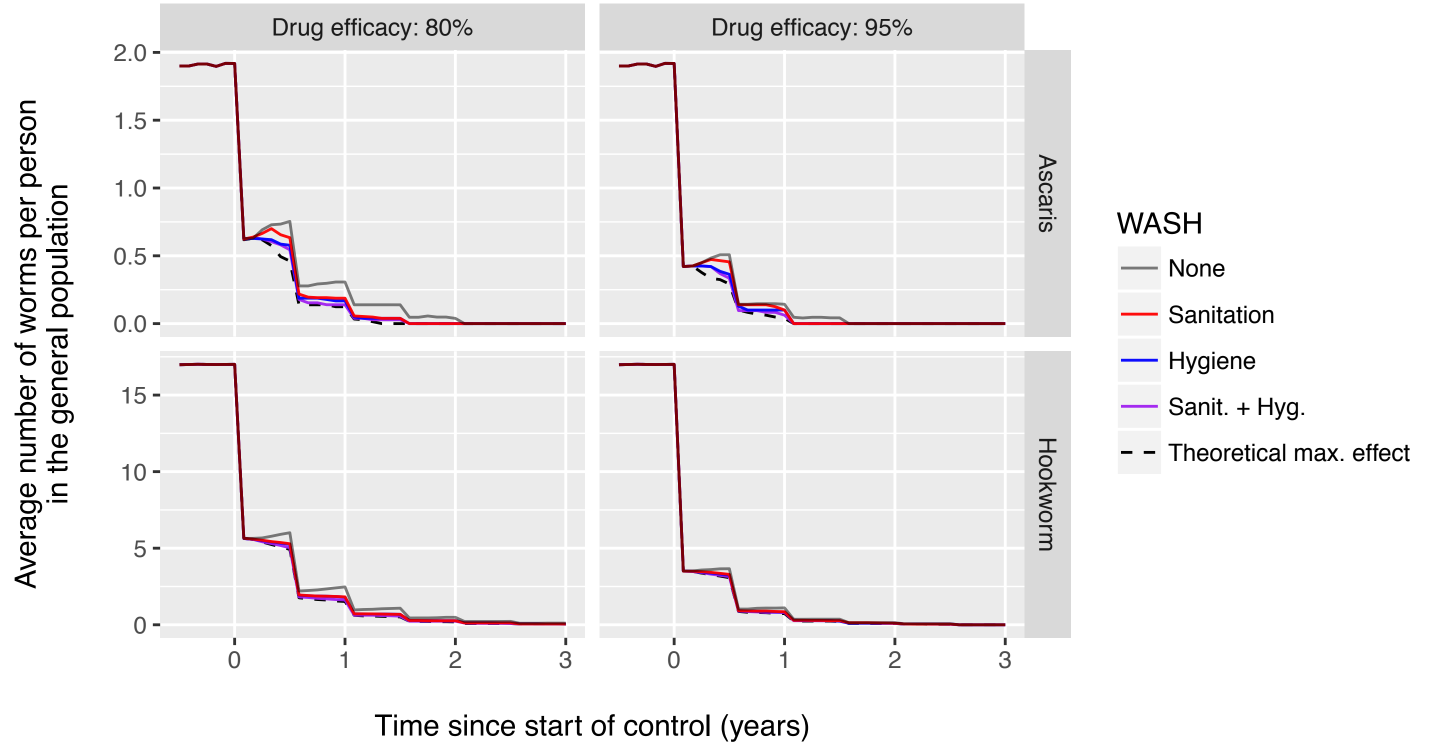

Drug efficacy: 80%
Drug efficacy: 95%
Ascaris
Hookworm
Average number of worms per person in the general population
Time since start of control (years)
2.0
1.5
1.0
0.5
0.0
15
10
5
0
0
1
2
3
WASH
None
Sanitation
Hygiene
Sanit. + Hyg.
Theoretical max. effect

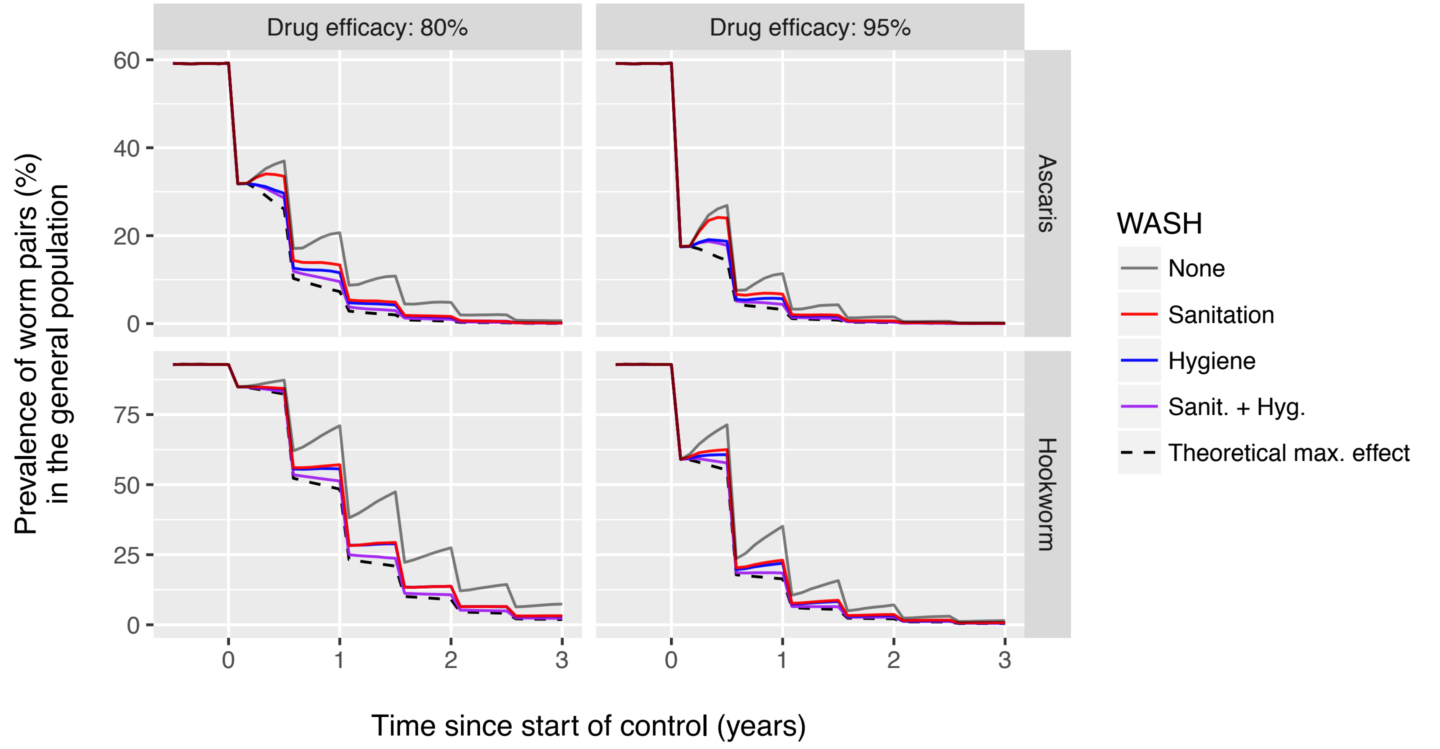

Drug efficacy: 80%
Drug efficacy: 95%
Ascaris
Hookworm
Prevalence of worm pairs (%)
in the general population
Time since start of control (years)
WASH
None
Sanitation
Hygiene
Sanit. + Hyg.
Theoretical max. effect
