# Supplementary material for: Predicted short and long-term impact of deworming and water, hygiene, and sanitation on transmission of soil-transmitted helminths
Source: PLoS Negl Trop Dis. 2018 Dec 6;12(12):e0006758. doi: 10.1371/journal.pntd.0006758 (PMC6283645; doi:10.1371/journal.pntd.0006758)
Supplement: S2 Fig — The figure represents a setting highly endemic for A. lumbricoides and hookworm (rows of panels) where semi-annual school-based deworming is implemented at 90% coverage of school-age children (SAC). Drug treatment is assumed to kill either 95% or 80% of worms in treated individuals (columns of panels). WASH interventions, if any, are assumed to be implemented at 70% uptake and 95% effectiveness. The dashed black line represents a theoretical scenario where WASH is implemented perfectly such that transmission stops from the first PCT round onwards. In this theoretical scenario, the small inter-treatment rebounds in infection levels in SAC are due to previously untreated pre-school age children entering the SAC age group. (PDF) [file pntd.0006758.s003.pdf]

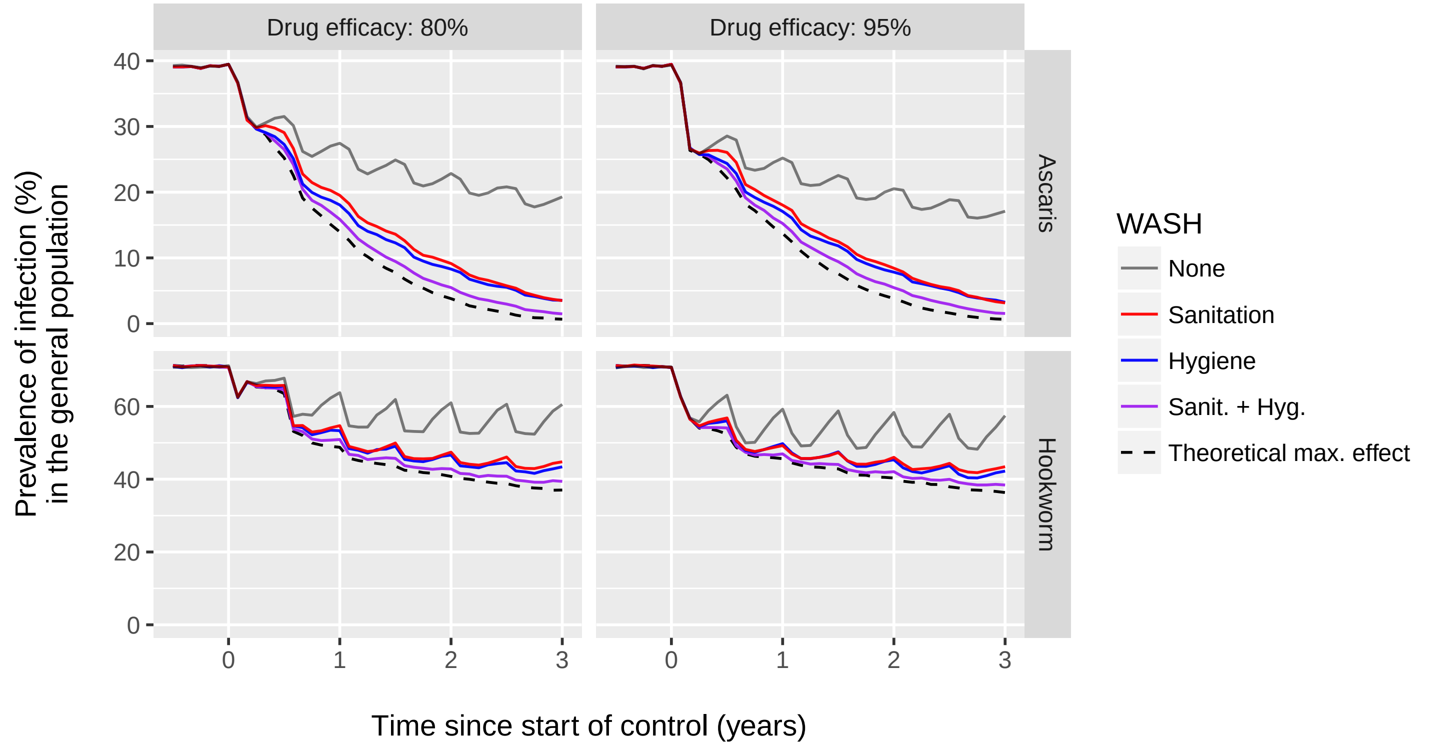

Drug efficacy: 80%
Drug efficacy: 95%
Ascaris
Hookworm
Prevalence of infection (%)
in the general population
Time since start of control (years)
WASH
None
Sanitation
Hygiene
Sanit. + Hyg.
Theoretical max. effect

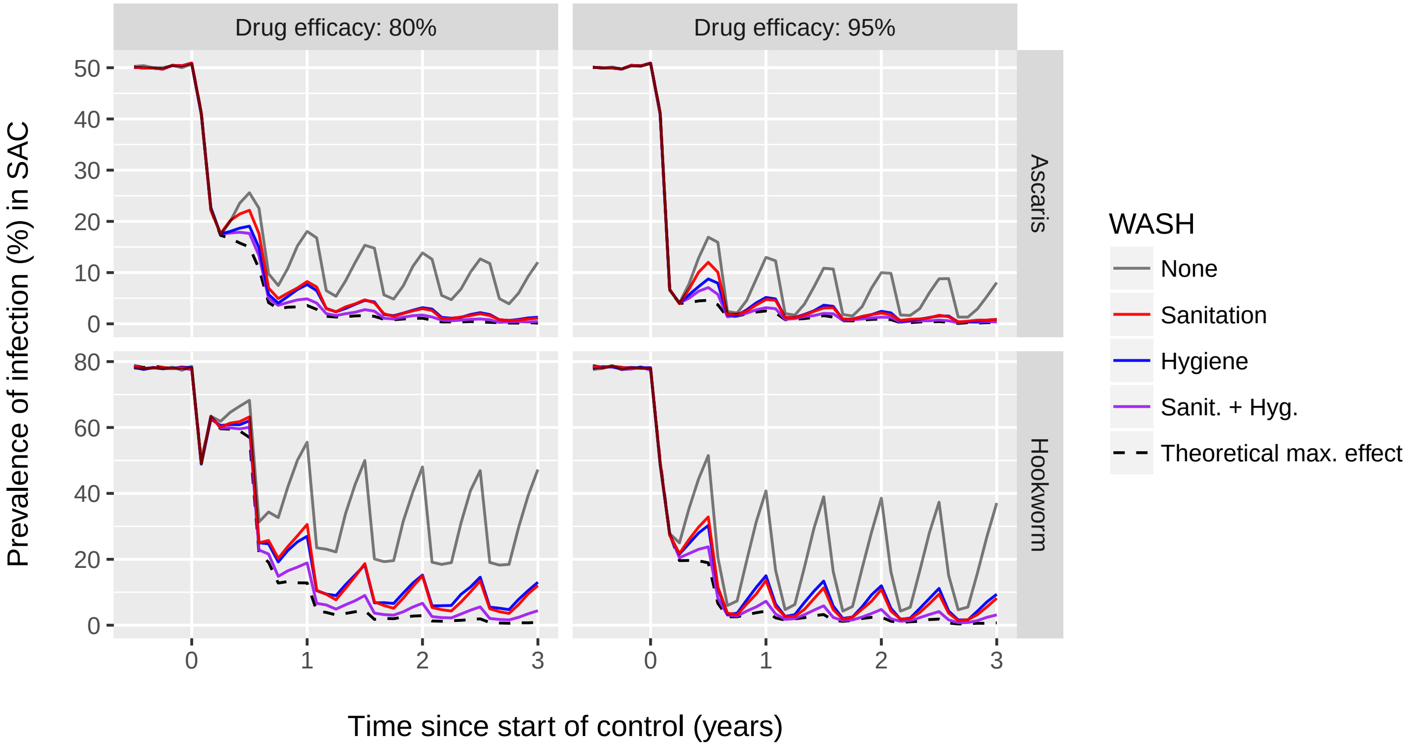

Drug efficacy: 80%
Drug efficacy: 95%
Ascaris
Hookworm
Prevalence of infection (%) in SAC
Time since start of control (years)
0
10
20
30
40
50
0
20
40
60
80
0
1
2
3
WASH
None
Sanitation
Hygiene
Sanit. + Hyg.
Theoretical max. effect

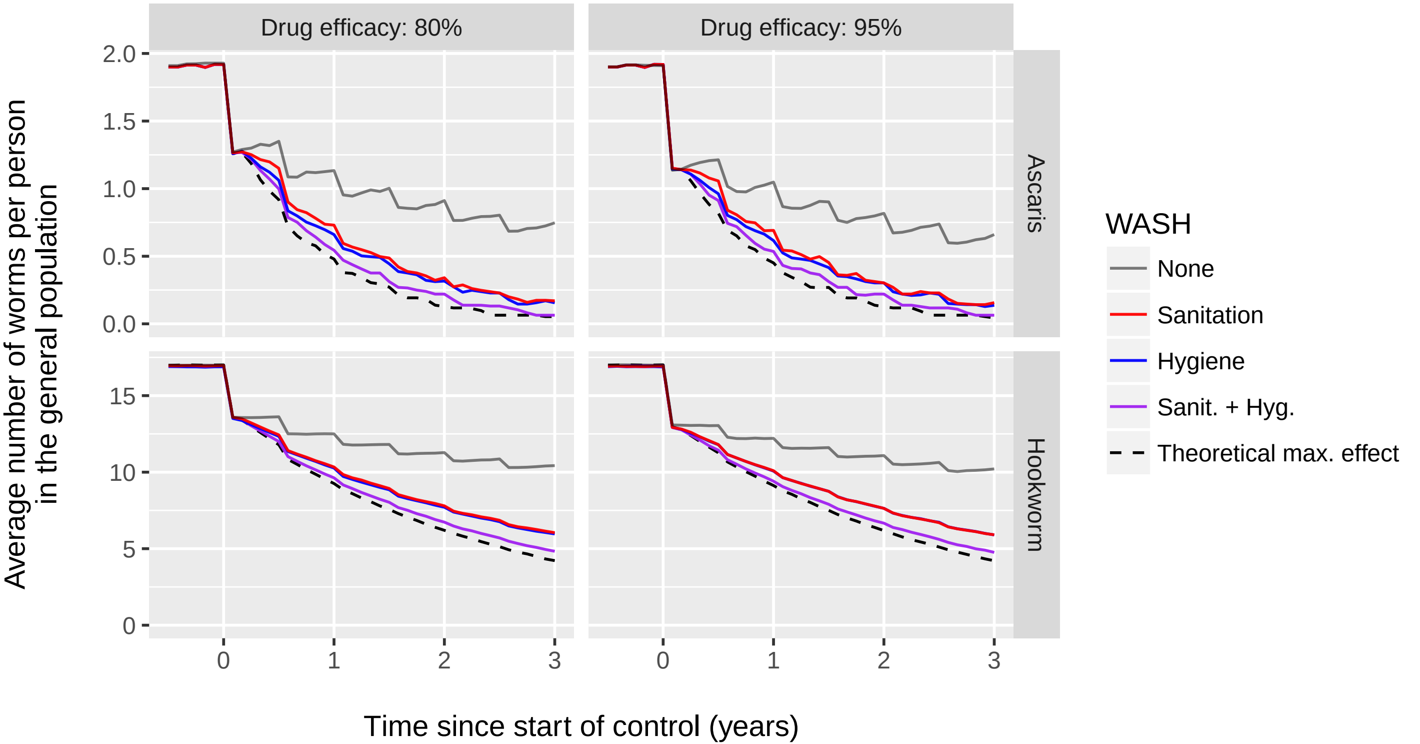

Drug efficacy: 80%
Drug efficacy: 95%
Ascaris
Hookworm
Average number of worms per person in the general population
Time since start of control (years)
WASH
None
Sanitation
Hygiene
Sanit. + Hyg.
Theoretical max. effect

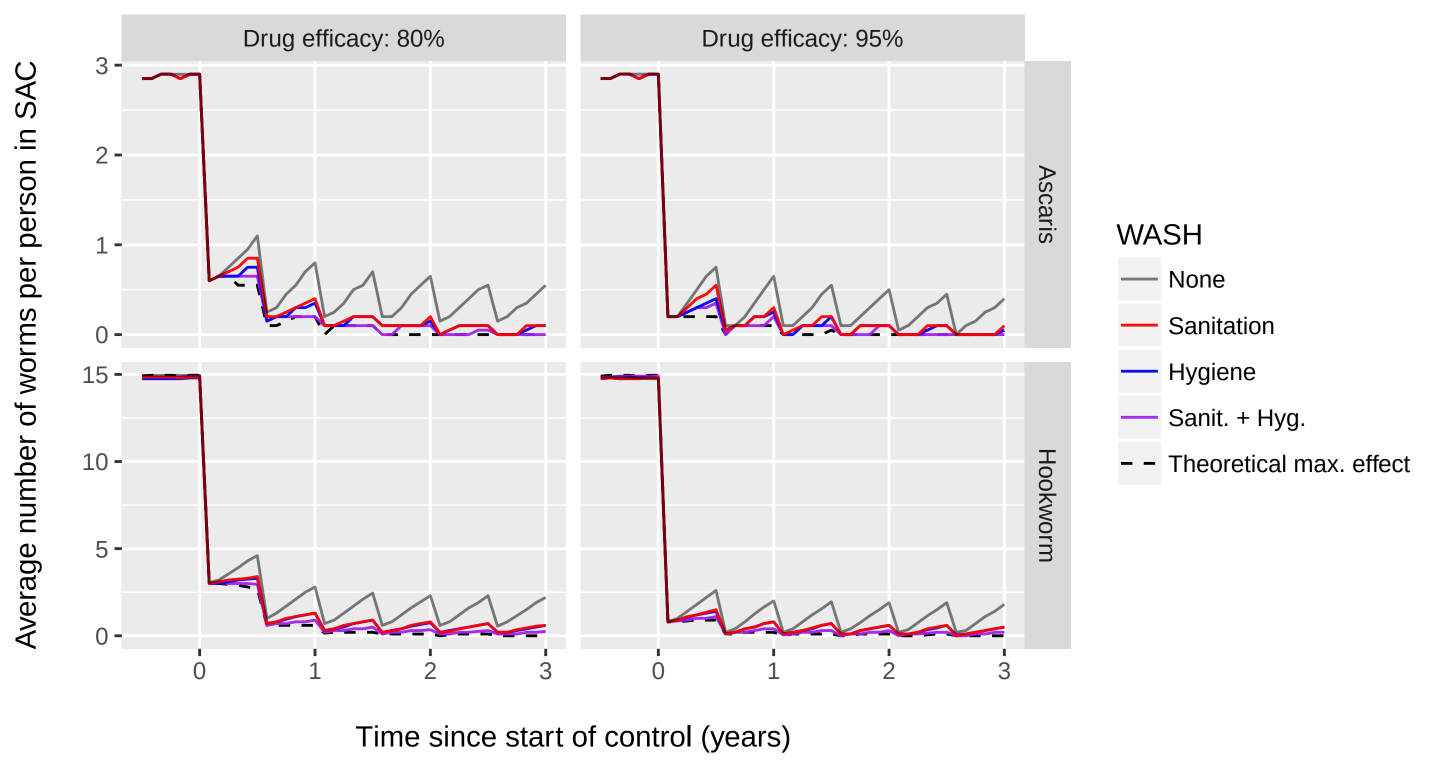

Drug efficacy: 80%
Drug efficacy: 95%
Ascaris
Hookworm
Average number of worms per person in SAC
Time since start of control (years)
WASH
None
Sanitation
Hygiene
Sanit. + Hyg.
Theoretical max. effect

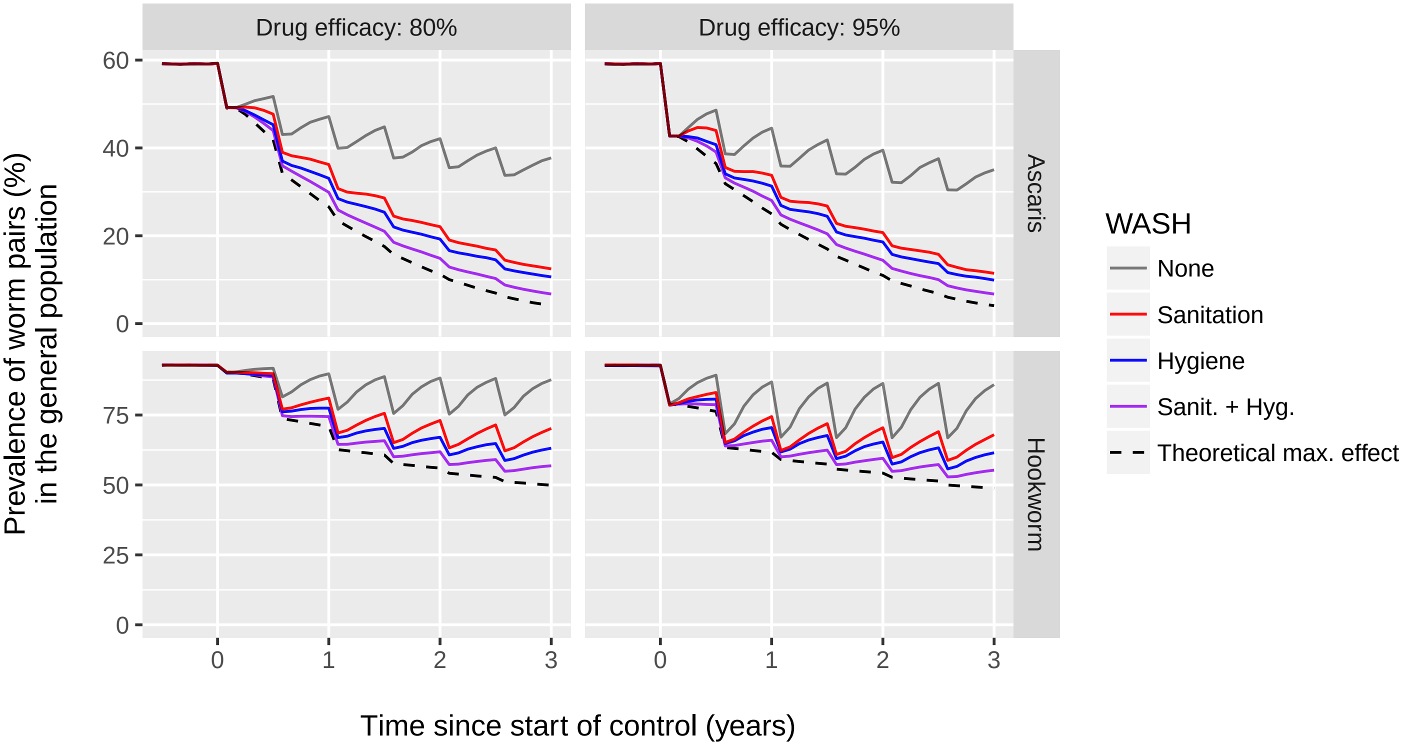

Drug efficacy: 80%
Drug efficacy: 95%
Ascaris
Hookworm
Prevalence of worm pairs (%)
in the general population
Time since start of control (years)
WASH
None
Sanitation
Hygiene
Sanit. + Hyg.
Theoretical max. effect

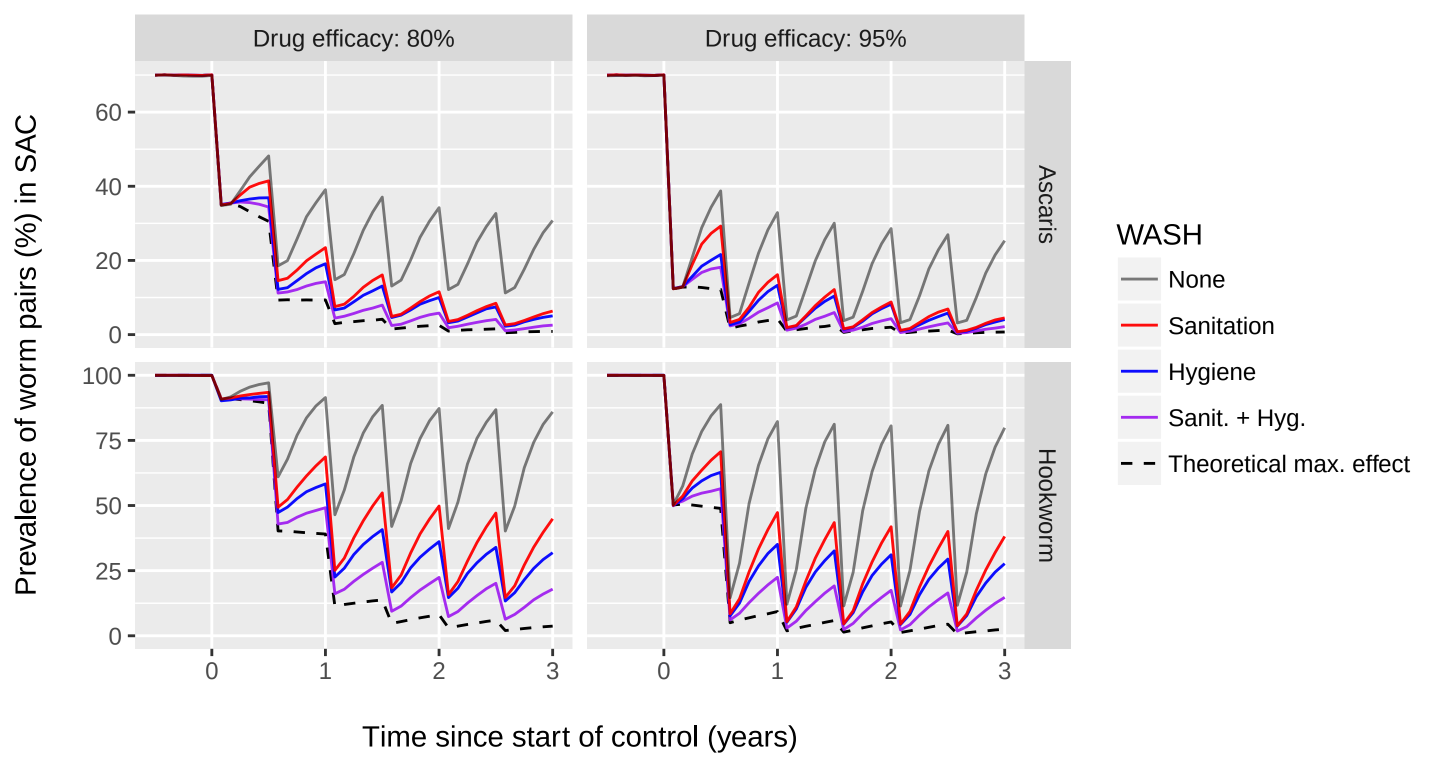

Drug efficacy: 80%
Drug efficacy: 95%
Ascaris
Hookworm
Prevalence of worm pairs (%) in SAC
0
20
40
60
0
25
50
75
100
0
1
2
3
Time since start of control (years)
WASH
None
Sanitation
Hygiene
Sanit. + Hyg.
Theoretical max. effect
