# Supplementary material for: Predicted short and long-term impact of deworming and water, hygiene, and sanitation on transmission of soil-transmitted helminths
Source: PLoS Negl Trop Dis. 2018 Dec 6;12(12):e0006758. doi: 10.1371/journal.pntd.0006758 (PMC6283645; doi:10.1371/journal.pntd.0006758)
Supplement: S3 Fig — Columns of panels refer to different WASH modalities and rows represent the three major soil-transmitted helminth species. Sanitation and hygiene interventions are assumed to reduce the contributions and exposure to the environmental reservoir of infection of individuals who take up the intervention, respectively. Coloured lines indicate different levels of uptake. Different levels of effectiveness (reduction of contribution and/or exposure to the environmental reservoir for individuals that take up the intervention) are indicated by solid vs. dashed vs. dashed-dotted lines. The dotted black line represents a theoretical scenario where the WASH modality (of that column of panels) is perfectly implemented and taken up, reducing exposure and/or contribution to transmission by 100% for all individuals. Predicted infection prevalences (y-axis) represent results from a single Kato-Katz slide. (PDF) [file pntd.0006758.s004.pdf]

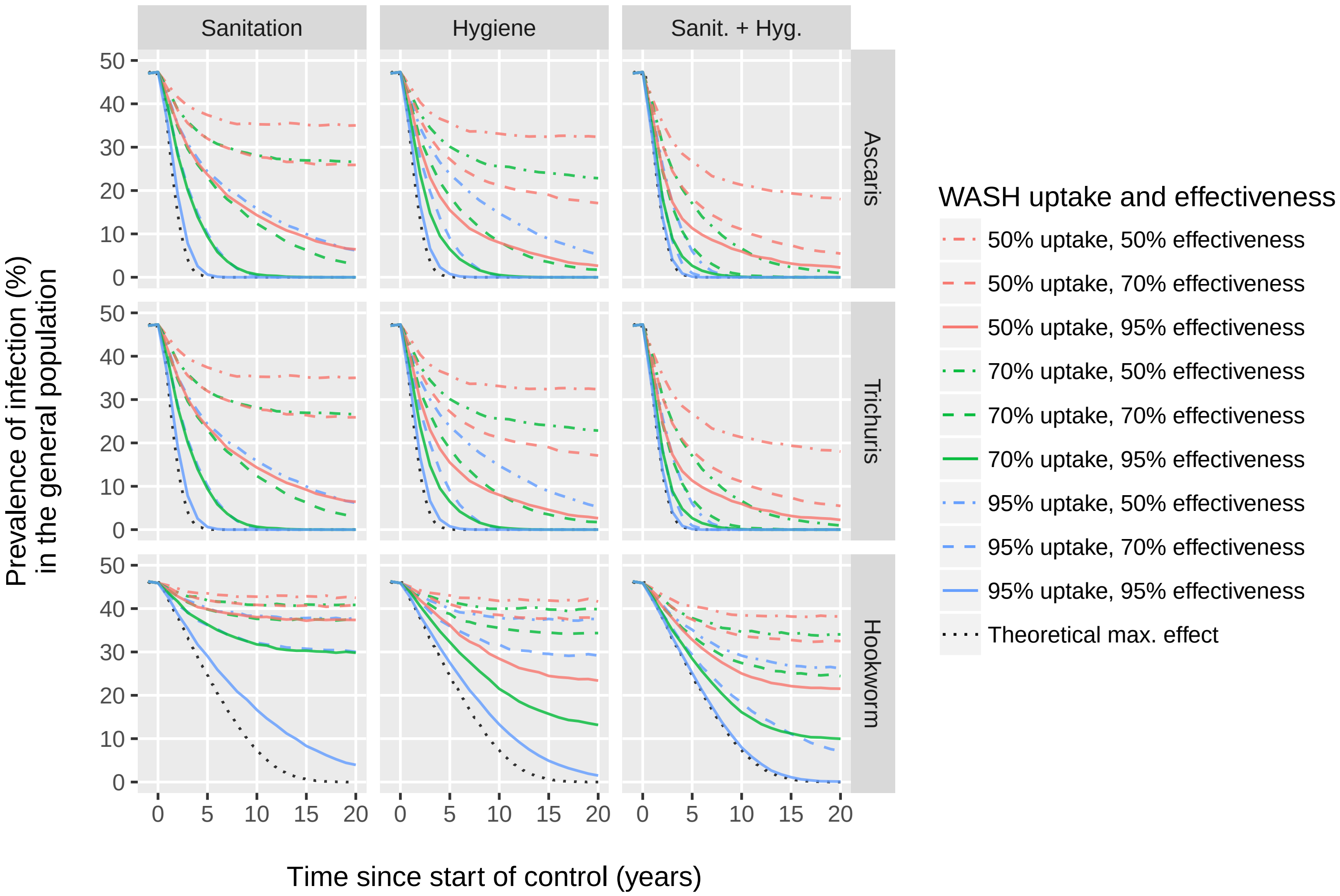

Sanitation
Hygiene
Sanit. + Hyg.
Ascaris
Trichuris
Hookworm
Prevalence of infection (%) in the general population
Time since start of control (years)
WASH uptake and effectiveness
50% uptake, 50% effectiveness
50% uptake, 70% effectiveness
50% uptake, 95% effectiveness
70% uptake, 50% effectiveness
70% uptake, 70% effectiveness
70% uptake, 95% effectiveness
95% uptake, 50% effectiveness
95% uptake, 70% effectiveness
95% uptake, 95% effectiveness
Theoretical max. effect
