# Supplementary material for: Predicted short and long-term impact of deworming and water, hygiene, and sanitation on transmission of soil-transmitted helminths
Source: PLoS Negl Trop Dis. 2018 Dec 6;12(12):e0006758. doi: 10.1371/journal.pntd.0006758 (PMC6283645; doi:10.1371/journal.pntd.0006758)
Supplement: S4 Fig — Lines represent the average of repeated simulations with random pre-control transmission conditions that followed a specific path through a decision tree for scaling down or stopping PCT as follows. Shaded areas represent the 2.5th and 97.5th percentiles of the stochastic variation in repeated simulations. For every single simulation, PCT was initiated at semi-annual or annual frequency (90% coverage of SAC or 75% of population of age 2 and above) if pre-control infection prevalence in SAC was >50% or between 20% and 50%, respectively; at midline (after 5 years of PCT, dashed vertical line) the PCT frequency was scaled down from semi-annual to annual if prevalence of infection in SAC was between 10% and 20%, and was scaled down to biennial (from either annual or semi-annual) if the prevalence of infection was between 1% and 10%. PCT was stopped if the midline prevalence of infection in SAC was <1%. WASH interventions were assumed start at midline and to be taken up by 70% of the population and to result in a 95% reduction in participating individuals’ contribution (sanitation) or exposure (hygiene) to the environmental reservoir of infection. Pre-control endemicity differs between the panels because the different paths through the decision tree are more likely in some transmission conditions than others (e.g. the decision to stop PCT only comes up in situations with relatively favourable (low) pre-control endemicity). Depicted average trends are the net effect of potential interruption of transmission in some simulations and bounce-back in others. (PDF) [file pntd.0006758.s005.pdf]

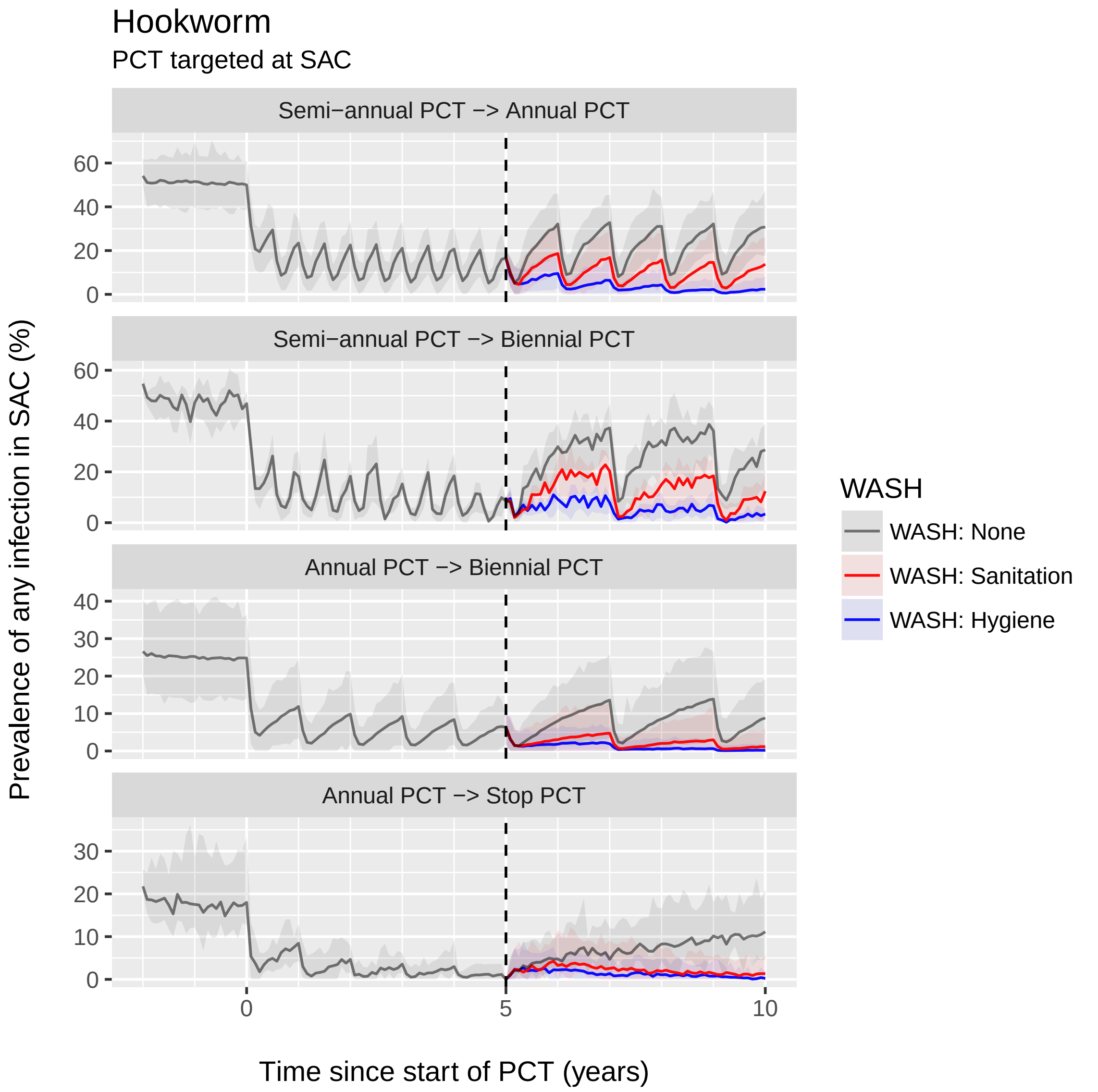

Hookworm
PCT targeted at SAC
Semi−annual PCT −> Annual PCT
Semi−annual PCT −> Biennial PCT
Annual PCT −> Biennial PCT
Annual PCT −> Stop PCT
Prevalence of any infection in SAC (%)
Time since start of PCT (years)
WASH
WASH: None
WASH: Sanitation
WASH: Hygiene

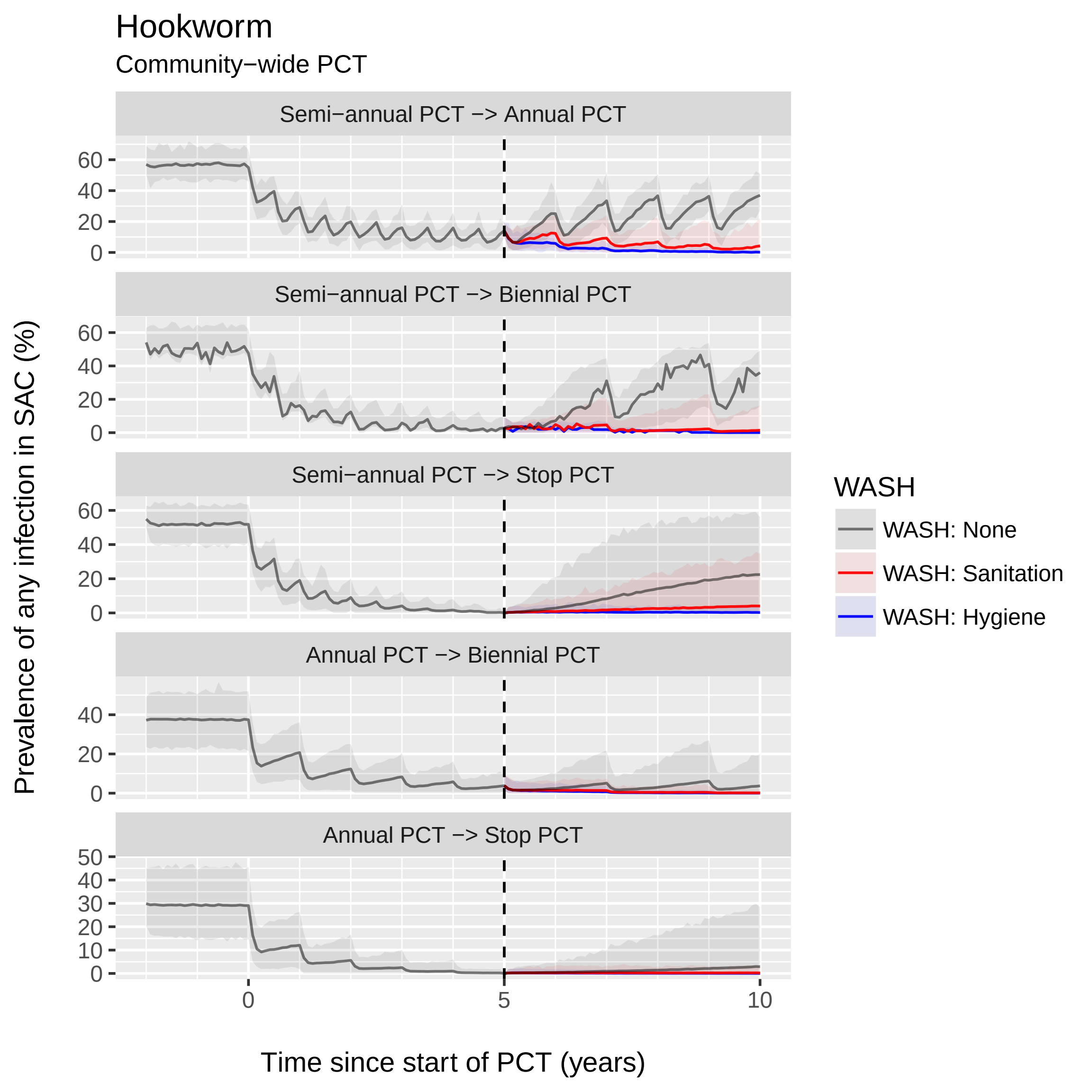
Hookworm
Community−wide PCT
Semi−annual PCT −> Annual PCT
Semi−annual PCT −> Biennial PCT
Semi−annual PCT −> Stop PCT
Annual PCT −> Biennial PCT
Annual PCT −> Stop PCT
Prevalence of any infection in SAC (%)
Time since start of PCT (years)
0
5
10
WASH
WASH: None
WASH: Sanitation
WASH: Hygiene

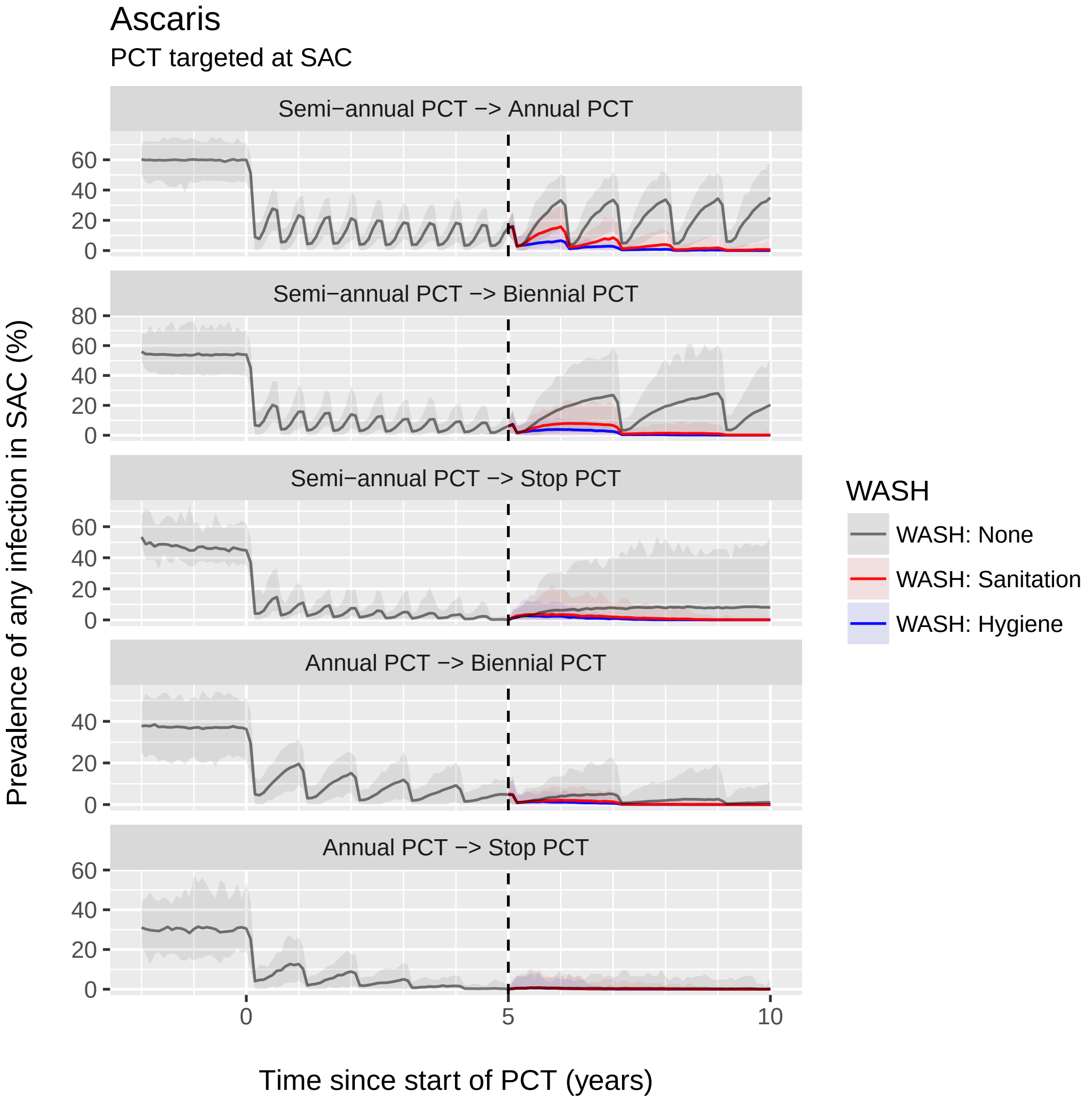

Ascaris
PCT targeted at SAC
Semi−annual PCT −> Annual PCT
Semi−annual PCT −> Biennial PCT
Semi−annual PCT −> Stop PCT
Annual PCT −> Biennial PCT
Annual PCT −> Stop PCT
Prevalence of any infection in SAC (%)
Time since start of PCT (years)
0
5
10
0
20
40
60
80
WASH
WASH: None
WASH: Sanitation
WASH: Hygiene

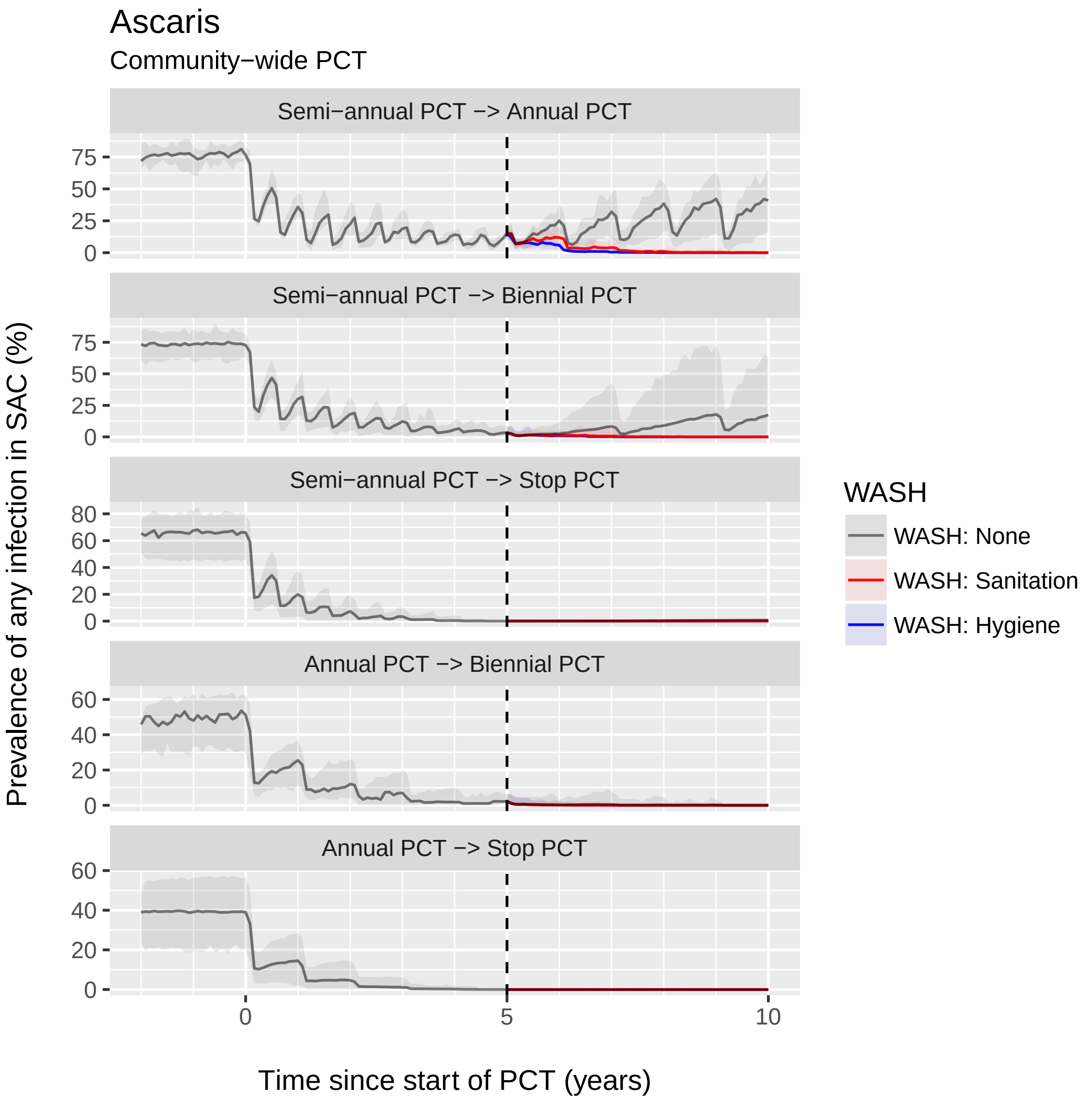

Ascaris
Community−wide PCT
Semi−annual PCT −> Annual PCT
Semi−annual PCT −> Biennial PCT
Semi−annual PCT −> Stop PCT
Annual PCT −> Biennial PCT
Annual PCT −> Stop PCT
Prevalence of any infection in SAC (%)
Time since start of PCT (years)
0
5
10
75
50
25
80
60
40
20
WASH
WASH: None
WASH: Sanitation
WASH: Hygiene

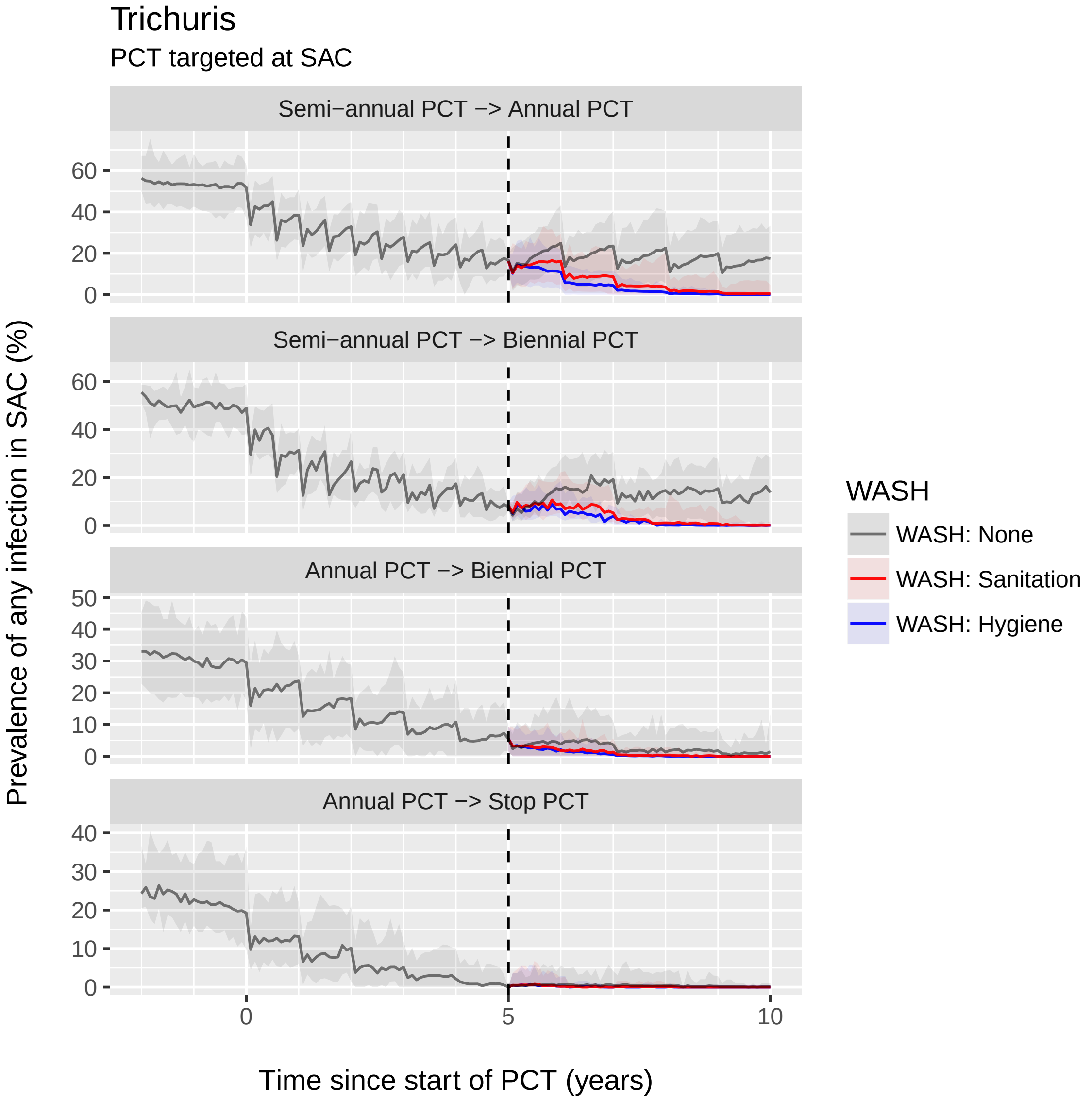
Trichuris
PCT targeted at SAC
Semi−annual PCT −> Annual PCT
Semi−annual PCT −> Biennial PCT
Annual PCT −> Biennial PCT
Annual PCT −> Stop PCT
Prevalence of any infection in SAC (%)
Time since start of PCT (years)
0
5
10
0
20
40
60
0
10
20
30
40
50
WASH
WASH: None
WASH: Sanitation
WASH: Hygiene

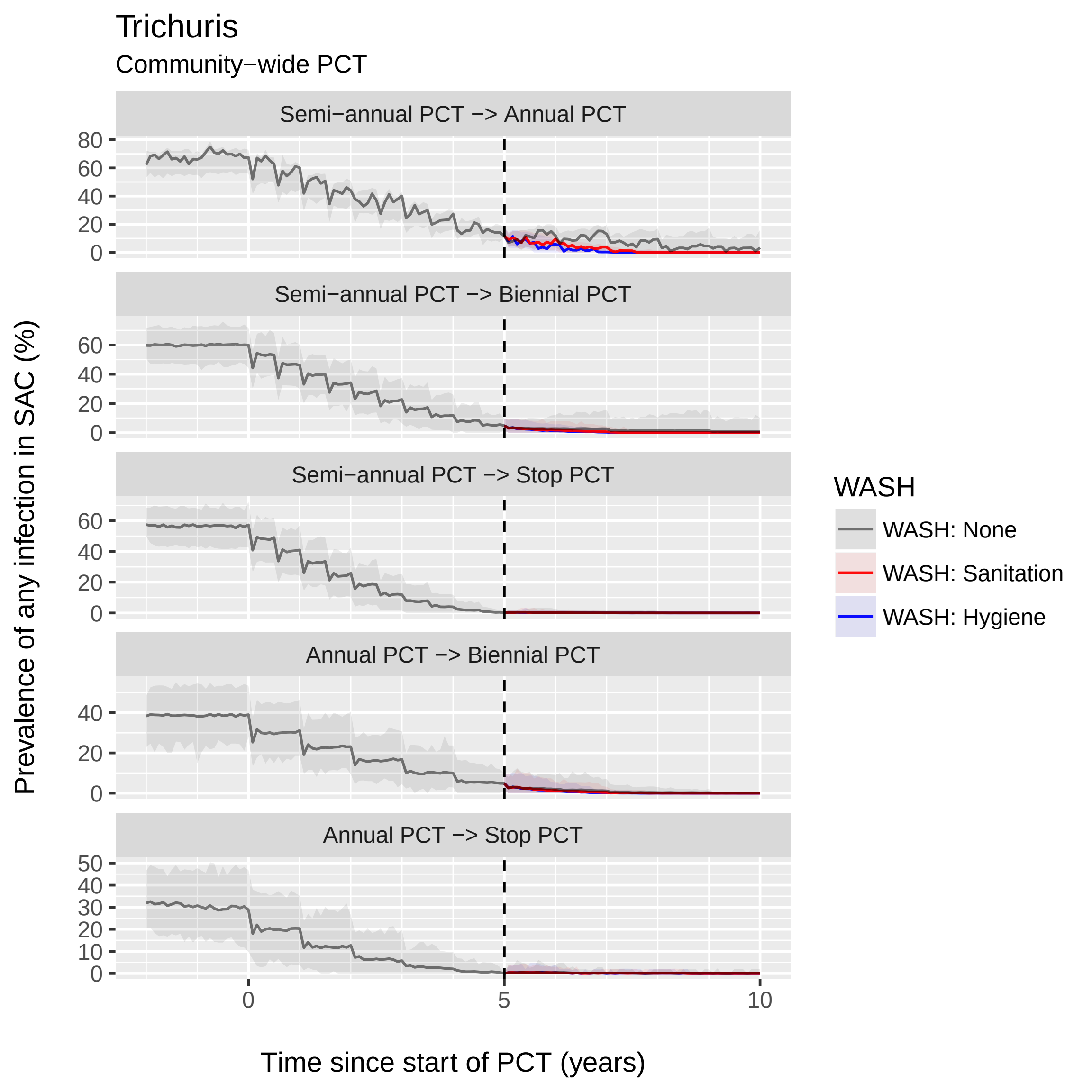

Trichuris
Community−wide PCT
Semi−annual PCT −> Annual PCT
Semi−annual PCT −> Biennial PCT
Semi−annual PCT −> Stop PCT
Annual PCT −> Biennial PCT
Annual PCT −> Stop PCT
Prevalence of any infection in SAC (%)
Time since start of PCT (years)
0
5
10
WASH
WASH: None
WASH: Sanitation
WASH: Hygiene
