# Supplementary figures and images for: Predicted short and long-term impact of deworming and water, hygiene, and sanitation on transmission of soil-transmitted helminths
Source: PLoS Negl Trop Dis. 2018 Dec 6;12(12):e0006758. doi: 10.1371/journal.pntd.0006758 (PMC6283645; doi:10.1371/journal.pntd.0006758)

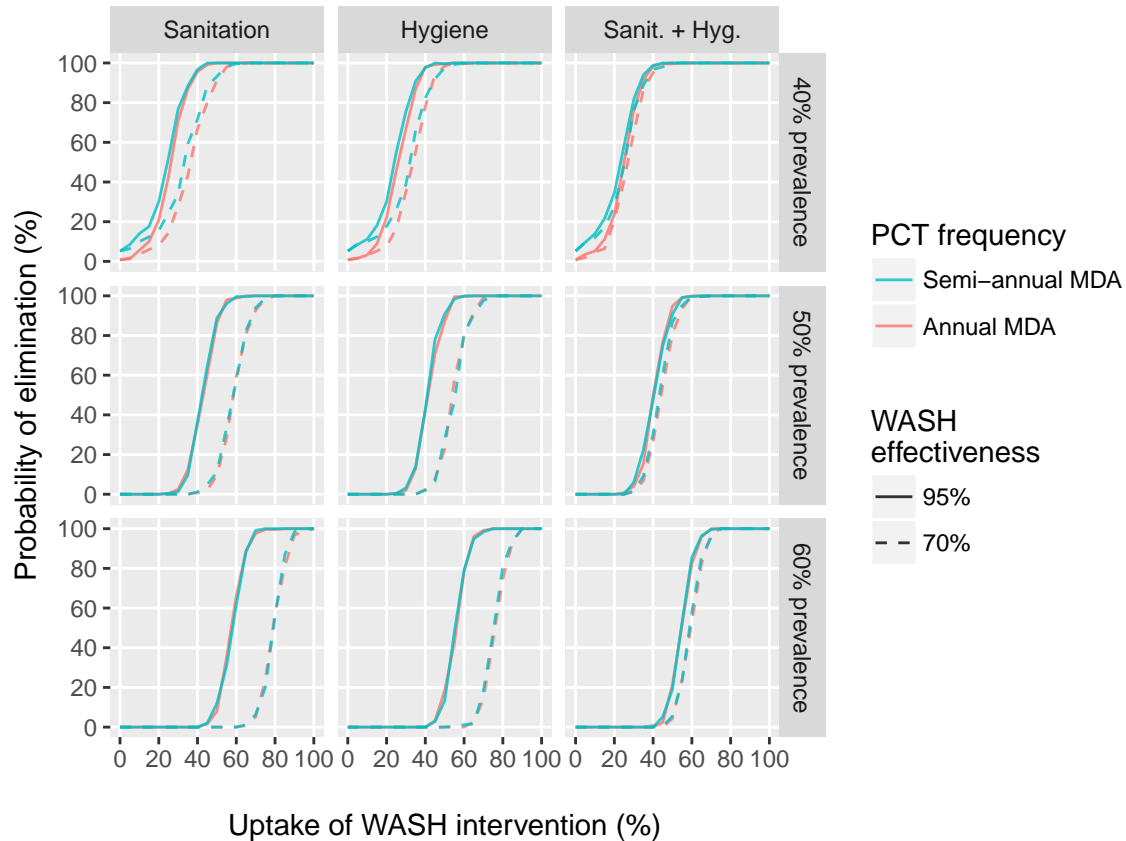

Supplement: S5 Fig — Rows of panels represent settings with different pre-control A. lumbricoides infection prevalence in the general population. For all three settings, we simulated five years of either annual (red lines) or semi-annual (green lines) school-based PCT with ALB, implemented at 90% coverage. After five years, PCT is stopped and one of three types of WASH interventions (columns) is simulated, which reduce participating individuals’ contribution (sanitation) and/or exposure (hygiene) to the environmental reservoir of infection by 70% or 95% (effectiveness), represented by solid and dashed lines, respectively. Uptake of WASH interventions (x-axis) is defined as the (random) proportion of people who take up the intervention. Uptake of sanitation and hygiene measures in the combined WASH intervention (third column) is assumed to be perfectly correlated within individuals. Elimination is defined as absence of any worms 50 years after stopping PCT in the simulated population of about 400 individuals. Lines for semi-annual and annual (red and green) are very close because neither strategy leads to a situation close to elimination after only 5 years of school-based PCT. (PDF) [file pntd.0006758.s006.pdf]

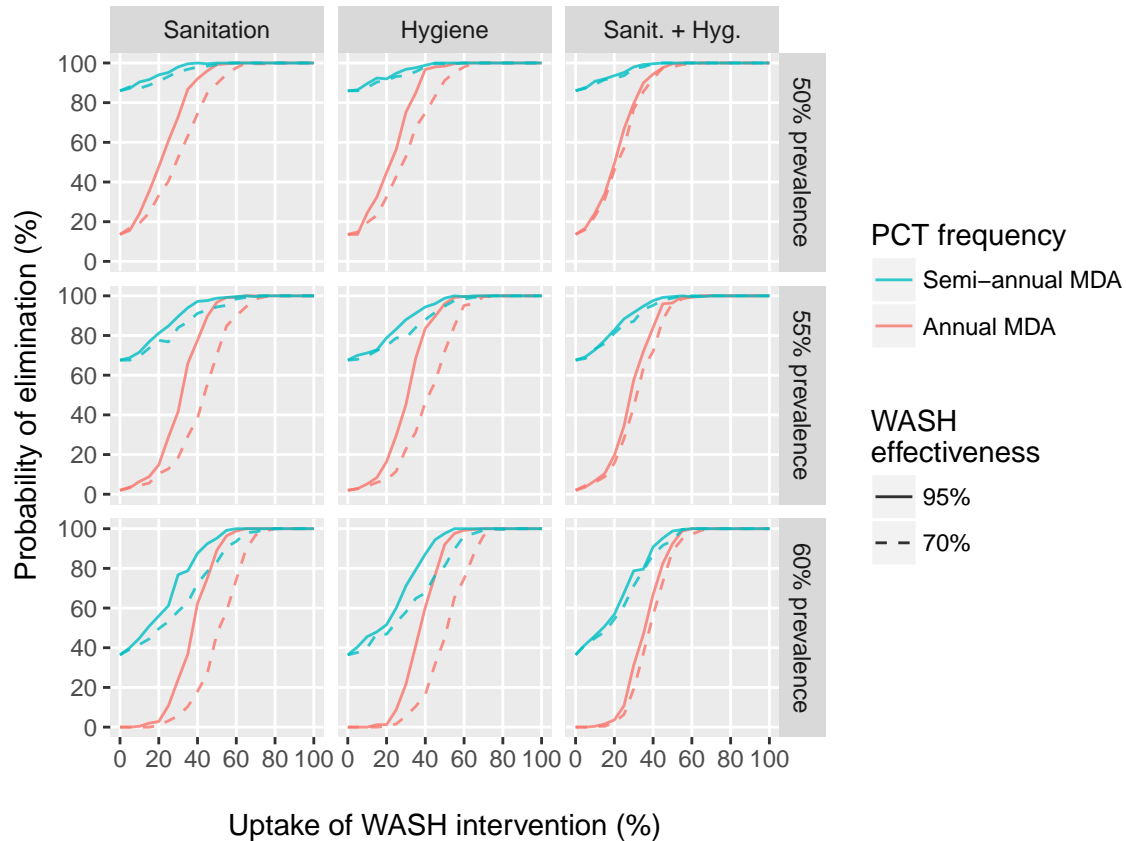

Supplement: S6 Fig — Rows of panels represent settings with different pre-control T. trichiura infection prevalence in the general population. For all three settings, we simulated five years of either annual (red lines) or semi-annual (green lines) community-wide PCT with ALB, implemented at 90% coverage. After five years, PCT is stopped and one of three types of WASH interventions (columns) is simulated, which reduce participating individuals’ contribution (sanitation) and/or exposure (hygiene) to the environmental reservoir of infection by 70% or 95% (effectiveness), represented by solid and dashed lines, respectively. Uptake of WASH interventions (x-axis) is defined as the (random) proportion of people who take up the intervention. Uptake of sanitation and hygiene measures in the combined WASH intervention (third column) is assumed to be perfectly correlated within individuals. Elimination is defined as absence of any worms 50 years after stopping PCT in the simulated population of about 400 individuals. (PDF) [file pntd.0006758.s007.pdf]
